# Sustained inhibition of CC-chemokine receptor-2 via intraarticular deposition of polymeric microplates in post-traumatic osteoarthritis

**DOI:** 10.1007/s13346-022-01235-1

**Published:** 2022-09-15

**Authors:** Huseyin Ozkan, Martina Di Francesco, Helen Willcockson, José Valdés-Fernández, Valentina Di Francesco, Froilán Granero-Moltó, Felipe Prósper, Paolo Decuzzi, Lara Longobardi

**Affiliations:** 1 Division of Rheumatology, Allergy and Immunology and the Thurston Arthritis Research Center, University of North Carolina-, Chapel Hill, 3300 Thurston Bowels Bldg, Campus, Box 7280, Chapel Hill, NC 27599 USA; 2 Laboratory of Nanotechnology for Precision Medicine, Fondazione Istituto Italiano Di Tecnologia, Genoa, Italy; 3 Cell Therapy Area, Clínica Universidad de Navarra, Pamplona, Spain; 4 Department of Orthopedic Surgery and Traumatology, Clínica Universidad de Navarra, Pamplona, Spain; 5 Program of Regenerative Medicine, Center for Applied Medical Research (CIMA), Universidad de Navarra, Pamplona, Spain; 6 Instituto de Investigacion Sanitaria de Navarra (IdiSNA), Pamplona, Spain; 7 Department of Hematology, Clínica Universidad de Navarra, Pamplona, Spain; 8 Program of Hemato-Oncology, Center for Applied Medical Research (CIMA), Universidad de Navarra, Pamplona, Spain

**Keywords:** Chemokines, Osteoarthritis, Local drug delivery, Polymeric microparticles, Murine osteoarthritis model

## Abstract

Posttraumatic osteoarthritis (PTOA) is mostly treated via corticosteroid administration, and total joint arthroplasty continues to be the sole effective intervention in severe conditions. To assess the therapeutic potential of CCR2 targeting in PTOA, we used biodegradable microplates (µPLs) to achieve a slow and sustained intraarticular release of the CCR2 inhibitor RS504393 into injured knees and followed joint damage during disease progression. RS504393**-**loaded µPLs (RS-µPLs) were fabricated via a template-replica molding technique. A mixture of poly(lactic-co-glycolic acid) (PLGA) and RS504393 was deposited into 20 × 10 μm (length × height) wells in a polyvinyl alcohol (PVA) square-patterned template. After physicochemical and toxicological characterizations, the RS504393 release profile from µPL was assessed in PBS buffer. C57BL/6 J male mice were subjected to destabilization of the medial meniscus (DMM)/sham surgery, and RS-µPLs (1 mg/kg) were administered intraarticularly 1 week postsurgery. Administrations were repeated at 4 and 7 weeks post-DMM. Drug free-µPLs (DF-µPLs) and saline injections were performed as controls. Mice were euthanized at 4 and 10 weeks post-DMM, corresponding to the early and severe PTOA stages, respectively. Knees were evaluated for cartilage structure score (ACS, H&E), matrix loss (safranin O score), osteophyte formation and maturation from cartilage to bone (cartilage quantification), and subchondral plate thickness. The RS-µPL architecture ensured the sustained release of CCR2 inhibitors over several weeks, with ~ 20% of RS504393 still available at 21 days. This prolonged release improved cartilage structure and reduced bone damage and synovial hyperplasia at both PTOA stages. Extracellular matrix loss was also attenuated, although with less efficacy. The results indicate that local sustained delivery is needed to optimize CCR2-targeted therapies.

**Graphical abstract:**

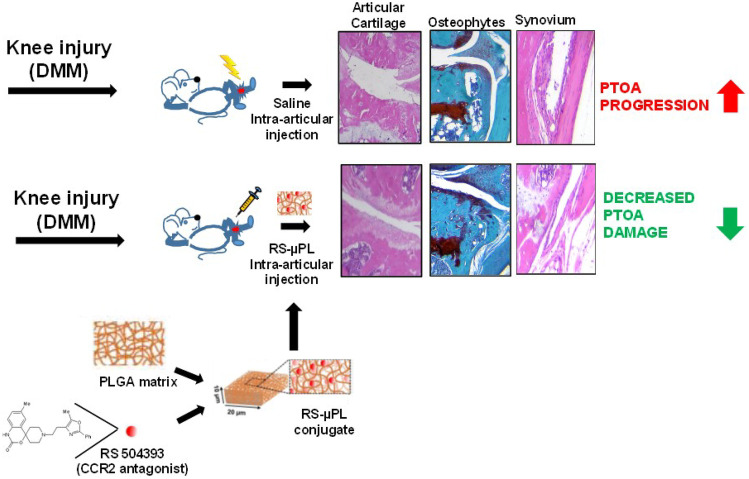

**Supplementary Information:**

The online version contains supplementary material available at 10.1007/s13346-022-01235-1.

## Introduction

Nearly half of patients sustaining significant joint damage will develop posttraumatic osteoarthritis (PTOA) [1]. Currently, PTOA treatments are mainly focused on attenuating pain and local inflammation via the chronic administration of corticosteroids. However, as extended exposure to systemic drug treatments can cause serious side effects, total joint arthroplasty continues to be the most effective approach in end-stage knee PTOA [2]. In this scenario, it is highly desirable to develop strategies that would allow a pharmaceutical compound to be protected from degradation and released in a sustained manner to the target tissue to mitigate systemic side effects while boosting therapeutic efficacy [3].

Mounting data have highlighted the importance of chemokines in acute knee injuries and osteoarthritis (OA) in both clinical [4–6] and preclinical studies [7–9]. The chemokine family comprises several protein members, including the chemokine (C–C motif) ligand type 2 (CCL2), which binds the same C–C chemokine receptor 2 (CCR2) [10], and they are known to be involved in the recruitment of immune cells at sites of inflammation [9, 11]. However, the relevance of the CCL2/CCR2 axis for potential PTOA therapies is not limited to mediating the inflammatory response accompanying disease progression but rather, unlike other biomarkers, is directly linked to cartilage and bone integrity, addressing the multitissue aspect of the disease. In addition to macrophages, CCR2 and CCL2 are expressed on chondrocytes, osteoblasts, and tendon fibroblasts, where they exert an important role in early tissue degeneration following injury [8, 12]. Our data obtained in human primary chondrocyte cultures demonstrated a temporal action of CCL2 on the expression of its own *Ccr2* receptor as well as on cartilage degradation markers (such as MMP1, MMP3, MMP13, and Timp1), activating specific MAP kinases (ERK and p38) and supporting the hypothesis that the CCL2/CCR2 axis might lead to cartilage dysregulation induced by OA [13]. Our animal studies documented CCL12/CCR2 activation in chondrocytes and osteoblasts immediately after injury and, in accordance with others, the protective action of early CCR2 blockade on cartilage and bone integrity [8]. However, the prolonged systemic administration of CCR2 antagonists has shown limited efficacy in attenuating joint structure changes, suggesting that high doses of CCR2 antagonists might produce undesired effects or alter CCR2 function in other cell systems, indirectly affecting PTOA therapy [8].

In the present study, we loaded polymeric, biodegradable microparticles, called microplates (µPLs), [14, 15] with the CCR2 antagonist RS504393 (RS-µPLs) to achieve its constant and slow release directly into the intraarticular space of PTOA knees. µPLs were synthetized using a template-replica molding fabrication technique that offers the opportunity to simultaneously and accurately tailor the particle size and shape (geometry) and mechanical properties, which are all relevant features in OA management [14–16]. The intraarticular deposition of RS-µPLs coupled with the sustained release of the CCR2 inhibitory molecules allowed us to considerably lower the RS504393 dosage and assess the therapeutic potential of CCR2 local targeting on PTOA progression; our results showed the efficacy of the local RS504393-µPL delivery, while the intraarticular injection of the free antagonist at the same doses was ineffective.

## Materials and methods

### Materials

Polydimethylsiloxane (PDMS) (Sylgard 184) and elastomer were purchased from Dow Corning (Midland, Michigan, USA). Poly(vinyl alcohol) (PVA, Mw 31,000 − 50,000), poly(D,L-lactide-coglycolide) acid (PLGA, lactide:glycolide 50:50, Mw 38,000 − 54,000), acetonitrile, the ATDC-5 cell line, and MTT assay were obtained from Sigma-Aldrich (Saint Louis, MO, USA). High-glucose Dulbecco’s modified Eagle’s minimal essential medium (DMEM)/F-12 GlutaMAX, penicillin, streptomycin, and heat-inactivated fetal bovine serum (FBS) were purchased from Gibco (Invitrogen Corporation, Giuliano Milanese, Milan, Italy). The CCR-2 antagonist RS-504393 was purchased from Biotechne/Tocris (Minneapolis, MN, USA). Tissues were fixed in paraformaldehyde (4%) in phosphate-buffered saline (Electron Microscopy Sciences, Hatfield, PA, USA). Histological stains included safranin O (1.5%) in ddH20 (Sigma; St. Louis MO, USA), fast green (0.04%) in ddH20 (Sigma; St. Louis, MO, USA), hematoxylin-modified Harris (Ricca; Arlington, TX, USA), and eosin Y (0.25%) w/v in 57% EtOH v/v (Ricca; Arlington, TX, USA). All reagents and other solvents were used as received.

### Methods

#### Fabrication and characterization of RS504393-loaded microplates

Prismatic particles (μPL) with a 20 × 20 μm^2^ square base and a 10 μm height were produced using a top-down approach, as previously reported [14, 16]. Briefly, a silicon template micropatterned with an ordered array of squared wells (20 μm by 10 μm) was used to obtain sacrificial polyvinyl alcohol (PVA) templates. Poly(lactic-co-glycolic acid) (PLGA, 15 mg) and RS504393 (1.25 mg/mL) were combined in a polymeric mixture that was accurately deposited within the template wells. The loaded PVA template was dissolved in water, and particles were collected via centrifugation [14, 16]. The ratio between the number of collected μPLs and the number of wells in the sacrificial template, defined as the fabrication yielding, was evaluated. Particle size, size distribution, and morphology were evaluated using scanning electron microscopy (SEM, Elios Nanolab 650, FEI), optical profilometry on a ZETA-20 optical profilometer (ZETA Instruments), and a Multisizer 4 COULTER particle counter (Beckman Coulter), as previously reported [14, 16].

#### Biopharmaceutical characterization of RS504393-loaded microplates

In addition, the loading (LE) and encapsulation efficiency (EE) of RS504393 into µPLs were also evaluated [14, 16]. The RS504393 release profile from RS-µPLs was studied in a confined microenvironment. Particles were placed in three Eppendorf tubes in 500 μL of PBS buffer (pH 7.4, 1 × , 37 ± 2 °C) under continuous rotation. At fixed time points, samples were collected and centrifuged (1717 g for 5 min). The pellet was resuspended in fresh PBS buffer, while the supernatant was analyzed using HPLC after the addition of an equal volume of acetonitrile [14]. RS504393 loading (LE) and RS-µPL encapsulation efficiency (EE) were evaluated as previously reported using high-performance liquid chromatography (HPLC) analysis.

1 $$\mathrm{LE }\left(\mathrm{\%}\right)= \frac{\mathrm{amount\; of \;RS }504393\mathrm{ \;in \;the \;particles }}{\mathrm{total \;mass \;of \;particles}}\times 100$$

2 $$E\mathrm{E }\left(\mathrm{\%}\right)= \frac{\mathrm{amount \;of \;RS }504393\mathrm{\; in \;the \;particles }}{\mathrm{initial\; amount \;of \;RS }504393\mathrm{\;used\; for \;particles \;synthesis}}\times 100$$

#### Evaluation of RS-μPL biocompatibility

Particle biocompatibility was studied in the murine chondrocyte cell line ATDC5 using the MTT assay. Cells were cultured in DMEM/F-12 and GlutaMAX medium supplemented with 10% FBS and 1% penicillin/streptomycin and maintained at 37 °C in 5% CO_2_. Twenty-four hours before the experiments, cells were seeded into 96-well plates at a density of 5 × 10^3^ cells per well [14, 15]. Then, they were incubated for 24, 48, and 72 h with different concentrations of free RS or RS-µPLs (namely, 5, 10, 20, and 40 μM of RS504393 in all cases) or an equivalent number of drug-free μPLs (DF-μPLs) to have the same number of particles for the different RS-µPL concentrations. Free RS504393 was dissolved in DMSO for its insolubility in the culturing media. After 24, 48, and 72 days of particle incubation with cells, MTT solution was added to each well, and after 4 h at 37 °C, the resulting formazan crystals were solubilized in ethanol. Their absorbance was quantified using a microplate spectrophotometer at 570 nm, with 650 nm as the reference wavelength (Tecan, Männedorf, Switzerland). The percentage of cell viability was assessed according to the following equation:

3 $$Cell \;Viability \left(\%\right)= \frac{ {Abs}_{t}}{ {Abs}_{c}}\times 100$$

where Abs_t_ and Abs_c_ are the absorbance of treated and control (untreated) cells, respectively.

#### Evaluation of particle interactions with cells

The interaction between μPLs and the ATDC5 cell line was studied using SEM and confocal microscopy. Briefly, ATDC5 cells (2 × 10^5^ cells) were seeded on glass slides for 24 h and then incubated with μPLs overnight. Samples were collected and treated as previously reported [14].

#### In vivo studies—animal model

All experiments described were conducted in 14-week-old male C57BL/6 mice housed in the same room. Mouse weight was between 24 and 28 g at the time of surgery. This study was performed in line with the principles of the Declaration of Helsinki. Animal use protocols were approved by the Animal Care and Use Committee of the University of North Carolina at Chapel Hill NC (IACUC # 20–075.0-C).

DMM/Sham surgery was induced as previously described [17–19] by making a vertical incision in the frontal part of the knee followed by the opening of the joint capsule. The meniscotibial ligament was then transected, and meniscus laxity was verified by the surgeon. In the sham group, the ligament was visualized and left untouched. The DMM model results in injury-induced OA more consistent with the human clinical disorder in that it allows loading during the slow progression of changes in cartilage and bone. DMM lesions progress in stages from early/mild (4 weeks postsurgery) to moderate (8 weeks) and then to severe OA (10–12 weeks) [10]. As such, the DMM-induced OA model is well suited to studies of the temporal progression of OA. For each experimental group, sham surgery and DMM were performed the same day by the same surgeon. On the day of surgery, a maximum of 10 surgeries/day were performed; to limit variability, mice of the same litter were allocated to the same experimental group (saline, DF-µPLs, or RS-µPLs and the same time point of euthanasia) and equally divided between sham surgery and DMM. Mice were assigned a sham surgery or DMM destination by an investigator different from the surgeon before the surgery occurred, and the surgeon was notified only after the joint capsule was opened. For anesthesia, isoflurane (1.5 to 3% by inhalation) was used, and postoperative analgesia (Buprenex, 0.05–0.1 mg/kg) was administered per UNC IACUC guidelines. Mice were monitored daily for the first week and then every other day.

#### In vivo studies—treatment

Free RS504393 (or vehicle, 0.1% DMSO), RS-μPLs, DF-μPLs or saline was administered intraarticularly one week after DMM/Sham. For studies on moderate/severe-OA stage (8/10 weeks post-DMM), injections were repeated every 3 weeks (wks) until euthanasia. Doses of free RS504393 or RS-μPLs were determined based on our previous DMM studies on RS504393 systemic administrations (4.0 mg/kg/day) [8]. To test the efficacy of lower doses (approximately 100-fold less than previous systemic administration) associated with local delivery, we used a total of 1 mg/kg for each administration. Knees from DMM/sham mice were dissected at 4 weeks postsurgery for early-stage OA and 8 weeks (for free RS504393) or 10 weeks postsurgery (for RS-μPLs) to analyze OA progression at the moderate/severe stage.

#### Histopathologic assessment of arthritis

Dissected knees were fixed in 4% paraformaldehyde overnight at RT. Following removal of fixative and rinsing in PBS, the knees were decalcified with Immunocal (StatLab, McKinney, TX) for 5–7 days and embedded in paraffin, and frontal sections (6 µm) were cut through the entire joint. Two sections per sample (one in the middle and one posterior) were stained with hematoxylin and eosin or safranin O/fast green, and images were taken with an Olympus BX51 microscope and a DP71 camera. Histological sections were assessed for OA grading using the AC structure score (ACS, scale 0–12) and the safranin O staining score (Saf-O, scale 0–12) [20]. The combination of ACS and Saf-O scores provides in-depth information regarding changes within the lesions for both articular cartilage structure and extracellular matrix integrity. The ACS focuses on AC structure, identifying fibrillations and clefts in the surface of the lamina, while Saf-O is tailored at identifying changes within the cell compartment and/or the extracellular matrix [20] (see Supplementary Methods). For both scoring systems, two adjacent midcoronal and posterior sections were stained with H&E (for ACS) or safranin O, and lesions were identified within the 4 compartments (medial and lateral tibial plateau and femoral condyles). The results were expressed as the average of scores in all quadrants in all sections.

Histomorphometric analyses using ImageJ were performed to quantify the subchondral bone area in the medial tibia [21] from mice used for Saf-O semiquantitative assessments [8]. Specifically, the subchondral plate of each section stained with Saf-O from all groups was encircled to measure the area. This area corresponds to the bone tissue between the calcified cartilage and the trabecular bone that surrounds the bone marrow regions. Sections were examined in a blinded fashion with ImageJ software [20]. Data were reported in square micrometers (sq µm) as the average of 4 sections for each mouse.

For osteophyte grading, we used the scoring system described by Little et al. developed for both osteophyte size (scale 0–3) and maturity, with the latter reflecting the osteophyte tissue composition (scale 0–3, with 3 defining bone predominance) [22]. Briefly, an osteophyte size score was obtained by assigning a score based on the size of osteophytes compared with adjacent cartilage (0 = none, 1 = small ~ the same thickness as the adjacent cartilage, 2 = medium ~ 1–3 × the thickness as the adjacent cartilage, and 3 = large > 3 × the thickness as the adjacent cartilage). The osteophyte maturity score reflects the tissue composition of osteophytes and is assessed by assigning a score as follows: 0 = none, 1 = predominantly cartilaginous, 2 = mixed cartilage and bone with active vascular invasion and endochondral ossification, and 3 = predominantly bone [21].

Histomorphometric analysis was performed to quantify the area of cartilage in the osteophytes and expressed as a percentage of the total osteophyte *area* (ImageJ) [21] (see Supplementary Methods). As osteophytes in the DMM are predominantly localized on the medial-tibial plateau, only this region was scored. One H&E-stained section/mouse from the posterior joint compartment was scored for synovial hyperplasia based on the scoring system described by Rowe et al. (scale of 0–3) [23]. The posterior region was used to avoid the area with the incision from the DMM. The details are as follows: 0 (none) = 1-cell layer in the synovium, 1 (mild) = 2–3 cell layers, 2 (moderate) = 4–5 cell layers, and 3 (severe) = 5 or more cell layers. The medial and lateral compartments of the joint were scored separately.

All scores were acquired in a blinded manner by three independent investigators, and the results are expressed as averages.

#### Statistics and sample size

For cell viability, five replicates were used for each experimental condition. This sample size achieves 95% power to detect a difference of at least 25 ± 10% (previous data) [14]. For in vivo experiments, we used 6 mice for each experimental group. The sample size achieves 95% power to detect a difference of at least 30% ± 10% (previous data) [8] using the pairwise multiple comparison test at 0.05 significance. No animals were excluded from the analysis. All group analyses were performed with ordinary two-way ANOVA (cell viability) or one-way ANOVA (in vivo studies), followed by Tukey’s post hoc test for multiple comparisons. For experiments with free RS504393 or vehicle, Student’s unpaired *t* tests were used at each time point post-DMM for both experimental sets. All outcomes, which were approximately normally distributed, were analyzed as continuous variables in their original scale. Statistical analyses were performed using GraphPad Prism Software (9.1.0). Statistical significance was set at *p* < 0.05. Data are expressed as the mean ± SD.

## Results

### RS504393-loaded microplate fabrication and physicochemical and biopharmaceutical properties

**Fig. 1:**
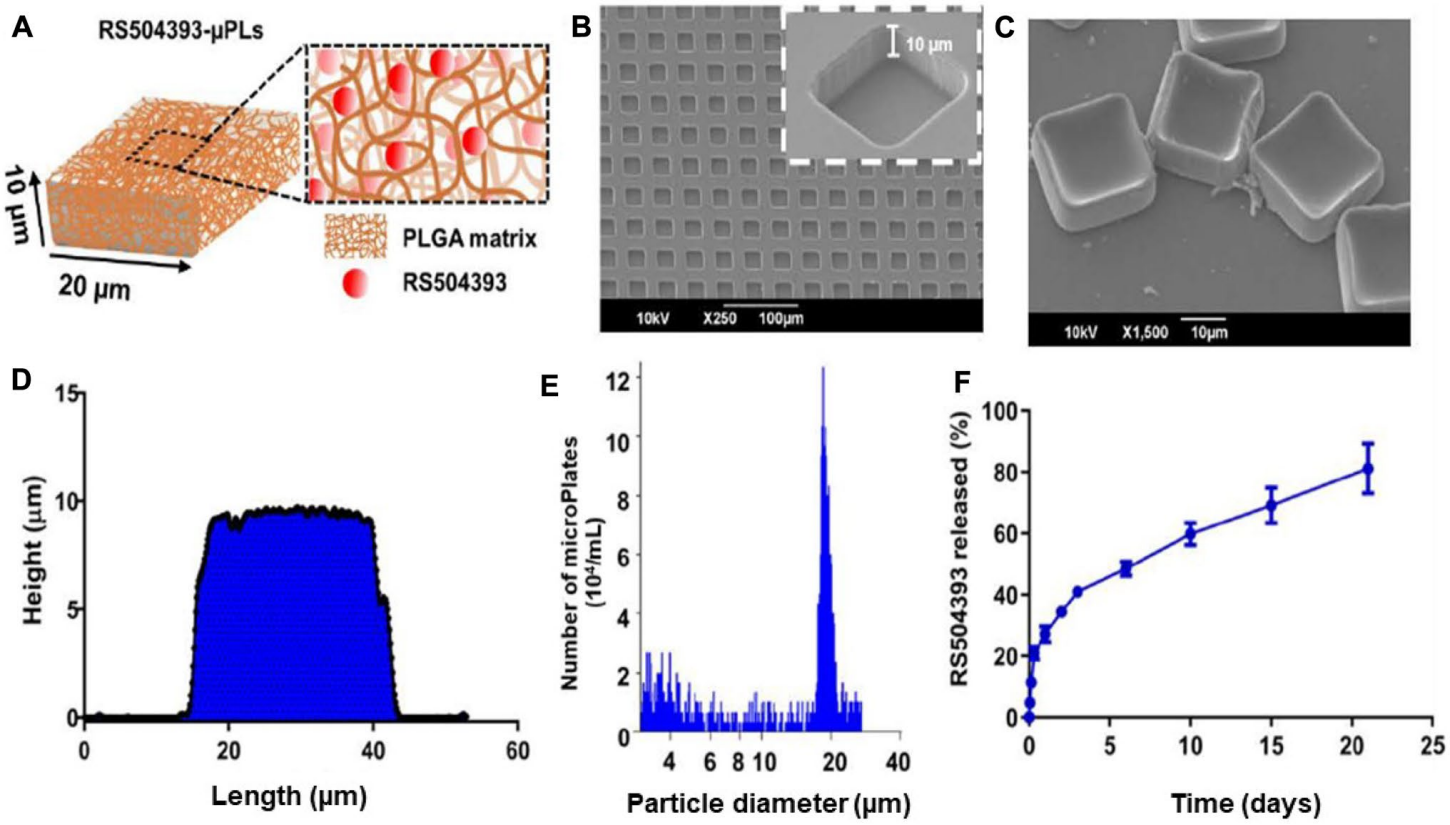
Physicochemical and biopharmaceutical characterizations of RS504393-loaded microplates (RS-µPLs). **A** Schematic representation of RS504393 dispersed in the poly(lactic-co-glycolic acid (PLGA) matrix of µPLs. **B**, **C** SEM analyses of a sacrificial polyvinyl alcohol (PVA) template and a purified set of µPLs. **D** Cross section of a µPL via profilometric analysis. **E** µPL size distribution and number evaluated via a Multisizer Coulter counter. **F** Release profile of RS504393 from µPLs under confined conditions (500 µL in PBS buffer = 500 µL) via high-performance liquid chromatography analysis (PLGA: 15 mg; RS504393: 1.25 mg/mL; *n* = 3 samples for each time point)

The microfabricated RS534393-loaded microplates (RS-µPL) are schematically shown in Fig. 1A. They were assembled employing lithographic techniques [14–16], returning particles with a characteristic square prismatic shape presenting a 20 µm edge length and a 10 µm height. Within the PLGA matrix (fibers), molecules of the CCR2 antagonist RS504393 (red dots) are uniformly dispersed. The RS-µPL size and shape correspond to the geometry of the wells in the original PVA template (Fig. 1B), as confirmed by scanning electron microscopy (SEM) images and profilometric analyses (Fig. 1C, D). Data from a Multisizer Coulter Counter returned a single sharp peak at approximately 20 μm, confirming the overall particle size and its homogenous distribution (Fig. 1E). The fabrication yield, defined as the ratio between the number of μPLs collected after the final purification step and the total number of wells in the original template (see Fig. 1B), was approximately 40%. This is in line with previously reported data from the authors [16].

The amounts of RS504393 per PVA template and per µPL were equal to 10.1 ± 1.03 μg and 27.9 ± 3.32 pg, respectively. The loading and encapsulation efficiency were 1.16 ± 0.23% and 15.6 ± 1.56%, respectively. The RS504393 release profile from RS-µPL was determined under confined conditions (500 µL, PBS) [14, 15]. RS504393 was released constantly into the medium, and after 21 days, 20% of the initial drug loading was still confined within the µPL structure (Fig. 1F, Supplementary Table S1). Based on this residual amount, it was estimated that an injection of RS-µPL every 3 weeks would suffice to achieve constant drug delivery in the joint.

**Fig. 2:**
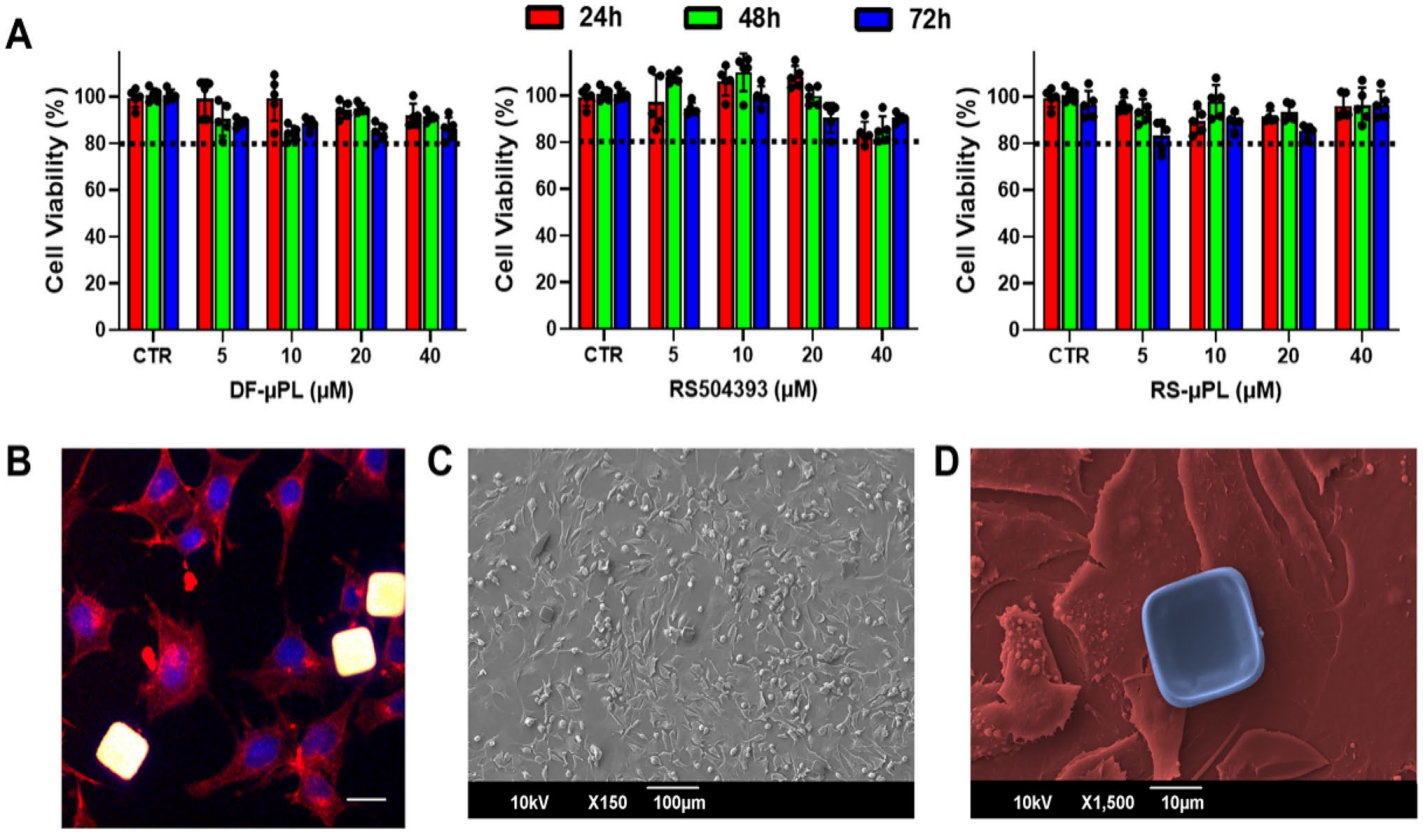
In vitro biocompatibility of µPL. **A** Viability analyses conducted on ATDC5 cells at 24, 48, and 72 h post-incubation with drug-free µPL (DF-µPL), free RS504393, and RS504393-loaded µPL (RS-µPL) at the same equivalent concentrations of RS504393 (5, 10, 20, and 40 μM). Data are presented as the mean ± standard deviation. **B**, **C** Representative confocal microscopy and scanning electron microscopy images, respectively, showing µPLs resting over a monolayer of ATDC5 cells. (A total of 2 ×10.^5^ cells were cultured on a glass slide for 24 h and then incubated with μPLs overnight)

RS-µPL showed an in vitro cell viability higher than 80% at any of the µPL or RS504393 concentrations for all tested time points (Fig. 2A), confirming that both the CCR2 inhibitor and the µPL have negligible effects on the viability of chondrocytes (Fig. 2A and Supplementary Table S2). Note that the number of particles to cells in the DF-μPL group (empty particles) matched that used for the RS-μPL (RS-loaded particles) group. Additionally, particles interacted with cells without being internalized, as confirmed by SEM and confocal analyses (Fig. 2B–D).

### RS504393-loaded microplates affect cartilage and bone damage in PTOA joints

We injected free RS534393 (or vehicle, 0.1% DMSO) intraarticularly at different time points after DMM/sham (Supplementary Fig. S1A) and followed PTOA damage on cartilage (AC structure and extracellular matrix, Supplementary Fig. S1B-G), bone (osteophyte formation, osteophyte maturity and subchondral bone thickness, Supplementary Fig. S2A-D), and synovium (lining cell hyperplasia, Supplementary Fig. S3A–C) at the early (4-week) and moderate (8-week) stages. The free antagonist was not able to rescue the degeneration of any of the tissues affected by PTOA at any of the experimental points.

**Fig. 3:**
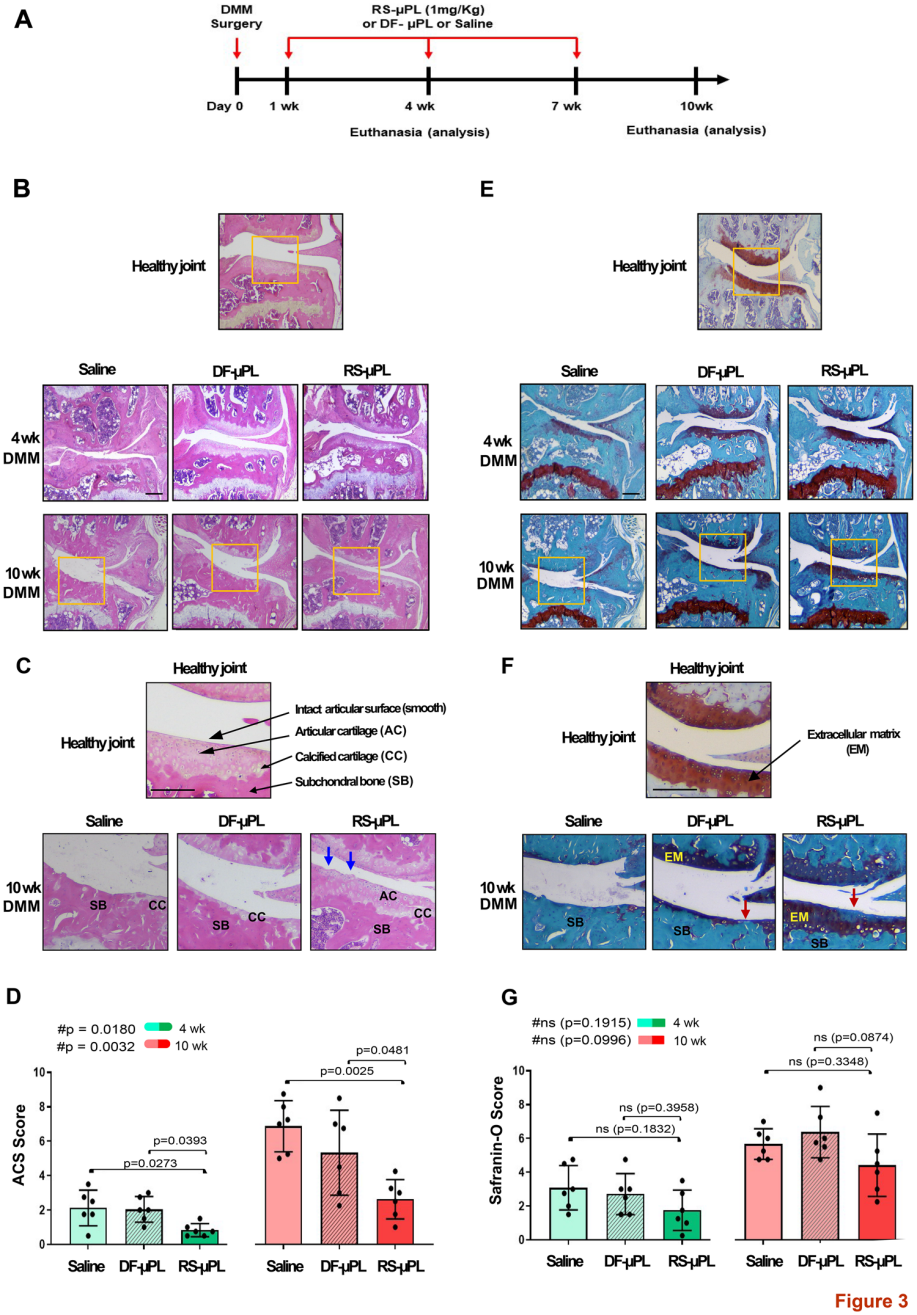
Histopathological evaluation of cartilage structure (ACS score) and extracellular matrix (safranin O score) of DMM mouse knees following intraarticular administration of RS-μPLs, DF-μPLs or saline solution. **A** Schematic view of the timeline for treatment and data evaluation. **B** H&E staining of the knee of DMM mice showing the medial compartment of a healthy joint as well as DMM joints injected intraarticularly with saline, drug-free μPLs (DF-μPLs) or RS504393-loaded μPLs (RS-μPLs), at the indicated times following surgery; images are representative of *n* = 6 for each time point. **C** Magnified images relative to the yellow square indicated in **B**; the healthy joint image shows the intact articular cartilage (AC) surface, the calcified cartilage (CC) below and the subchondral bone (SB), with the latter showing more intense pink staining. Magnifications of the DMM knees are from the severe PTOA stage (10 weeks) and show complete absence of cartilage in the saline sample, which is partially rescued in the RS-μPL-treated samples; however, the AC lamina is irregular, and a few clefts are still present (blue arrows). A thin layer of CC is visible in the DF-μPLs. **D** ACS semiquantitative score (0–12 scale) of DMM knees at the time points indicated reflecting the structure of the articular surface (lamina); the grading accounts for both the depth and extension of the damage. The results are expressed as the average of 4 quadrants (medial and lateral tibial plateau, medial and lateral femoral condyles); *n* = 6 mice for each experimental point. **E** Safranin O/Fast green staining of the knee of DMM mice showing the medial compartment of a healthy joint as well as DMM joints injected intraarticularly with saline, drug-free μPLs (DF-μPLs) or RS504393-loaded μPLs (RS-μPLs) at the indicated times following surgery images are representative of *n* = 6 for each time point. **F** Magnified images relative to the yellow square indicated in **E**; the healthy joint image shows thick red safranin O staining, indicating the extracellular matrix (EM), while single cells are visible in darker color, and a green/blue color identifies the subchondral bone. Magnifications of the DMM knees are from the severe PTOA stage (10 weeks) and show complete absence of safranin O staining, which is partially rescued in the RS-μPL-treated samples, although loss of cells and red staining is visible on the joint surface (red arrows). A thin layer of matrix is visible in the DF-μPLs. **G** Safranin O semiquantitative score (0–12 scale) of DMM knees at the time point indicated, reflecting loss of EM; the grading accounts for both the depth and extension of the damage. The results are expressed as the average of 4 quadrants as described above; *n* = 6 mice for each experimental point. The graphs represent the mean ± standard deviation. # indicates one-way ANOVA at each time point; multiple comparison values by Tukey’s post-hoc test are indicated in each graph. Scale bars of the images are 100 µm

To determine whether a more constant and stable release of RS504393 in the injured knees would improve PTOA outcomes, we injected RS-µPL, DF-µPL or saline into DMM/sham knees at the same time points used for free RS504393. We analyzed joint damage at the early OA stage (4 weeks) and extended the long-term analysis to a more severe stage (10 weeks postsurgery) (Fig. 3A). As shown in Fig. 3B–D, CCR2 blockade in DMM knees significantly decreased ACS scores at both the early and severe OA stages compared with DF-µPLs (*p* = 0.0393, 0.0481) or saline (*p* = 0.0273, 0.0025). A decreased Saf-O score was also seen in DMM knees injected with RS-µPLs at both OA stages compared with DF-µPLs and saline, but the changes were less evident and did not reach statistical significance (Fig. 3E–G and Supplementary Table S3). No differences in ACS and Saf-O scores were detected among the shams in any of the experimental groups, including free RS504393, at either OA stage (Supplementary Fig. S4).

**Fig. 4:**
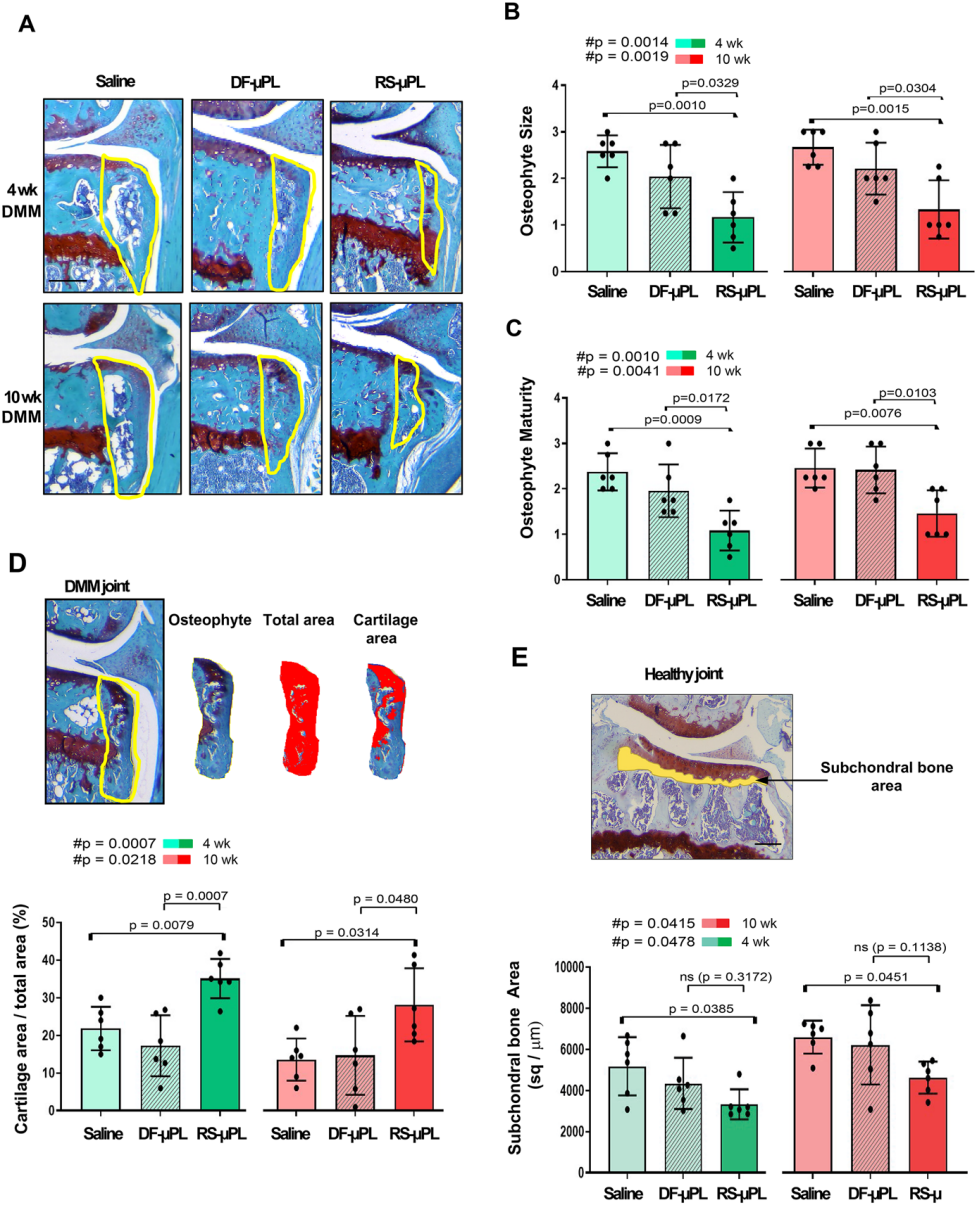
Osteophyte assessment and subchondral bone quantification of DMM mouse knees following intraarticular administration of RS-μPL, DF- μPL or saline solution. **A** Safranin O/Fast green staining of osteophyte formations in the medial tibial compartment of DMM mice injected intraarticularly with saline, drug-free μPLs (DF-μPLs) or RS504393-loaded μPLs (RS-μPLs) at the indicated times; osteophytes are circled in the images, representing new cartilage/bone formations emerging at the level of the lateral tibia; images are representative of *n* = 6 for each of the experimental points described. **B** The osteophyte size score at the time points indicated represents a semiquantitative grading of the size of the newly formed osteophytes (scale 0–3). The results are expressed as the average of 4 quadrants (medial and lateral tibial plateau, medial and lateral femoral condyles); *n* = 6 mice for each experimental point. **C** The osteophyte maturity score (scale 0–3) at the time point indicated represents a semiquantitative grading of the amount of bone tissue in the osteophytes. The results are expressed as the average of 4 quadrants (medial and lateral tibial plateau, medial and lateral femoral condyles); *n* = 6 mice for each experimental point. **D** Quantification of the area of cartilage in the osteophytes was calculated by histomorphometric analysis and expressed as a percentage of the total osteophyte area at each experimental time point (circled in yellow in the example image and cut for measurement purposes); *n* = 6 mice for each experimental point. **E** Quantification of the subchondral plate area (sq/µm) of the tibia medial plateau of DMM knees by histomorphometric analysis at the time point indicated; anatomically, the subchondral bone is defined between the calcified cartilage and the trabecular bone that surrounds the bone marrow regions (highlighted in yellow in the example image); *n* = 6 mice for each experimental point. The graphs represent the mean ± standard deviation. # indicates one-way ANOVA at each time point; multiple comparison values by Tukey’s post hoc test are indicated in each graph. Scale bars of the images are 100 µm

We also evaluated changes in DMM-induced osteophyte formation and subchondral bone thickness in DF-µPL-injected mice (Fig. 4 and Supplementary Table S3). As shown in Fig. 4A, a constant intraarticular inhibition of CCR2 affects osteophyte formation compared with DF-µPLs and saline, diminishing their size (Fig. 4B) and modifying their tissue composition (cartilage vs. bone) at both OA stages, as defined by the maturity score (Fig. 4C) and osteophyte cartilage quantification (Fig. 4D). We rarely observed osteophytes in sham controls (Supplementary Fig. S4A); therefore, shams were not graded. When evaluating the subchondral bone, we found that CCR2 targeting reduced the DMM-induced thickness at both stages when compared with saline-injected samples; however, changes were less evident when compared with the DF-µPL treated samples and were not significant (Fig. 4E). No changes in subchondral bone thickness were found in the shams among all groups (Supplementary Fig. S5).

Of note, injections with DF-µPLs appeared to lead to a slight improvement in the ACS score, osteophyte size and maturity when compared with saline injections, although none of the differences were significant (Supplementary Table S3).

**Fig. 5:**
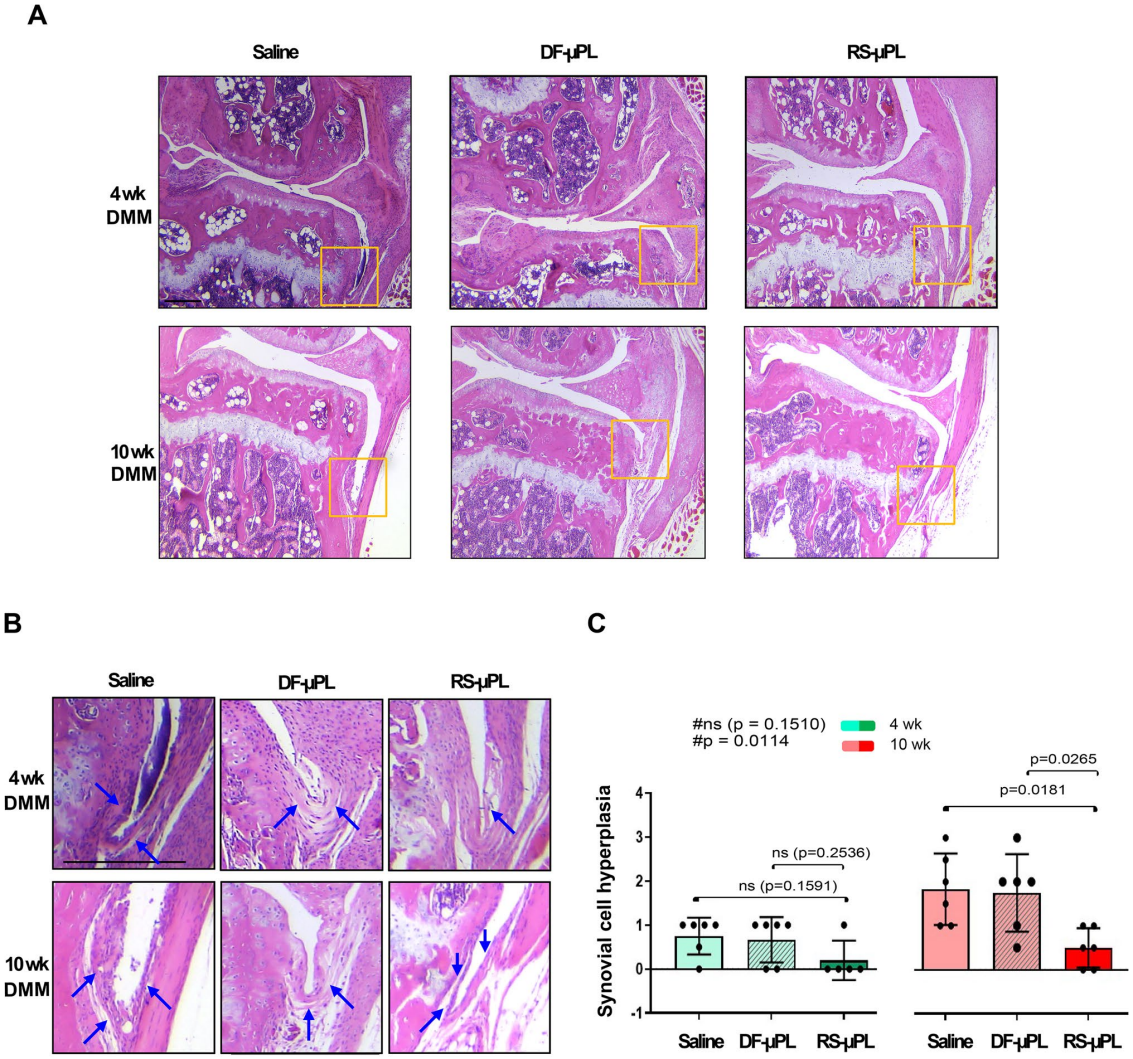
Synovial hyperplasia assessment of DMM mouse knees following intraarticular administration of RS-μPLs, DF-μPLs or saline solution.** A** H&E staining of the synovial lining cells of DMM mice injected intraarticularly with saline, drug-free μPLs (DF-μPLs) or RS504393-loaded μPLs (RS-μPLs) at the indicated times following surgery; images are representative of *n* = 6 for each time point. **B** Magnified images relative to the yellow square indicated in **A**; images show the thickness of the synovium in all samples; specifically, the saline samples and DF-μPL-injected samples at the severe stages (10 weeks) are characterized by multiple cell layers (more than 2 cells thick); RS-μPL-injected samples show a thinner synovium (blue arrows). **C** Synovial hyperplasia scores (scale 0–3) at the time point indicated reflecting the thickness of the synovium (number of cell layers in the thickest point). Scores reflect the highest grade of the medial and lateral tibial plateau, medial and lateral femoral condyles; *n* = *6* mice for each experimental point. The graphs represent the mean ± standard deviation. # indicates one-way ANOVA at each time point; multiple comparison values by Tukey’s post hoc test are indicated in each graph. Scale bars of the images are 100 µm

CCR2 has an established role in inflammation [9, 11]; therefore, we next analyzed whether its local targeting could affect synovial hyperplasia mediated by injury. We measured the thickness of the lining cells of the synovium, and we found that a constant intraarticular release of RS504393 reduced the hyperplasia, with effects that were more prominent at the severe stage (Fig. 5A–C). No differences in synovial hyperplasia were detected in any of the shams at any of the experimental points (Supplementary Fig. S6).

## Discussion

CCR2 and CCL2 have been extensively studied in inflammatory diseases, particularly in rheumatoid arthritis. Different molecules have been developed to target either the receptor (monoclonal antibody MC-21 [24] and small molecules such as RS504393 and cenicriviroc [7, 8, 25]) or the ligand (Nox-E36) in different pathologies [26, 27]. Specific to PTOA, preclinical studies in rodents have tested the ability of the CCR2 inhibitor RS504393 to reduce histologic OA severity and pain, leveraging its small size and high specificity in antagonizing the CCR2 receptor [7, 8, 28]. In this study, we analyzed whether local targeting of CCR2 using RS504393 had beneficial effects on PTOA degeneration using the DMM model. Our results demonstrated that local intraarticular injections of free RS504393 (1 mg/kg, every 3 weeks) were not able to ameliorate cartilage and bone damage induced by DMM or synovial hyperplasia (Supplemental Fig. S1, S2 and S3), although others reported efficacy on knee hyperalgesia (intraarticular, 0.5 mg/kg) when measures were assessed in the first 4 h post-injection [28]. These data suggest that while the short permanence of the drug into the joint space can alleviate pain by directly stimulating the intraarticular sensory afferents, it might not be sufficient to prevent long-term damage to the joint structure. Therefore, after reformulating the CCR2 antagonist RS504393 into homogeneous microparticles (RS-µPLs) and demonstrating their ability to release the therapeutic compound slowly and in a sustained fashion, we tested RS-µPLs in the DMM model at doses 100-fold lower than previously reported for systemic treatments, with 1 injection every 3 weeks [8]. Moreover, as previously demonstrated, µPLs were retained for almost 1 month after a single intraarticular injection in the knee of a murine PTOA model [14, 15]. It is also important to observe that this prototypical formulation of RS504393 into µPLs provides a significant improvement in joint health despite the relatively low encapsulation efficiency. This appears to be in line with similar drug delivery systems previously developed for diverse medical applications by the authors [14, 16, 29, 30] and with the commercially available ZILRETTA microparticles, returning a nominal drug load of 25% for the synthetic corticosteroid triamcinolone acetonide [31, 32]. Indeed, a higher EE of up to 80% could be obtained by using drug conjugates, such as dexamethasone palmitate, as recently documented by Fattal and his group [33].

Our results showed that local continuous CCR2 inhibition in the mouse knee during PTOA has a beneficial effect on the AC lamina, preserving its structure at both early and late OA stages (ACS score, Fig. 3B–D). We also dissected the contribution of CCR2 to the integrity of the extracellular matrix followed by injury (Saf-O score); although slightly reduced damage was detectable by CCR2 blockade, the variability among the samples was high, and the results did not reach statistical significance (Fig. 3E–G). This difference might reveal a clinical significance, reflecting the activation of distinctive catabolic responses mediated by CCR2. Our previous in vitro data on human chondrocytes showed that exogenous treatments with CCL2 led to gene upregulation of different cartilage degradation markers mediated by distinct signaling pathways [34]. This would suggest that distinct CCR2-mediated pathways might affect different aspects of cartilage pathology, and certain pathways might potentially be more impactful on specific cartilage features. This information opens new perspectives for the use of selective molecules that could optimize CCR2 targeting in different types of OA, such as aging- or inflammatory-mediated OA, as well as in different arthritic diseases, where a variety of cartilage catabolic events might occur at various disease stages.

With respect to joint degeneration following trauma, Appleton et al. previously reported a protective role for CCR2 antagonism in PTOA by in vivo pharmacologic inhibition using RS504393 (given systemically at 200 nmol/kg/hour by a miniosmotic pump) in a rat anterior cruciate ligament transection (ACLT) model coupled with meniscectomy, although the effect was more pronounced at later OA stages [7]. Our previous mouse studies in a DMM model demonstrated that systemic transient targeting of CCR2 early after trauma (RS504393 added to the drinking water, from 1 to 4 weeks postinjury) led to sustained protective action on cartilage and bone that extended through severe PTOA stages [8]. However, in contrast with data in the rat ACLT model, prolonged and continuous systemic administration of the CCR2 antagonist (from 1 to 12 weeks) showed limited efficacy in attenuating joint structure changes. This discrepancy in the timing of CCR2 action may be due to the more severe rat model involving knee ACL transection and partial meniscectomy [7]. Indeed, unlike DMM, ACL transection may be associated with patellar maltracking or dislocation of the patella, with much faster progression of joint degeneration and additional intraarticular inflammation [35]. Therefore, sustained inflammation following severe injury may play a significant role in CCR2-mediated bone changes and may reflect different temporal stages compared with DMM-induced OA. Taken together, these studies suggest that a systemic approach to CCR2 targeting might alter CCR2 function in other cell systems outside the joints and highlight the need for local targeting for PTOA therapeutic purposes [8].

Bone involvement is a hallmark of OA [36–38], and bone damage occurs early following DMM, with a chondro/osteophyte appearance and increased subchondral bone thickness by 2 weeks postinjury [8]. CCR2 has been proven to have a direct role in modulating both skeletal repair and bone remodeling [39]. Accordingly, in this study, we found that intraarticular CCR2 targeting was able to affect PTOA bone changes, decreasing osteophyte formation and delaying ossification as well as reducing the thickness of the subchondral plate (Fig. 4B–E).

In contrast to systemic CCR2 blockade, where prolonged exposure to the antagonist resulted in a diminished efficiency on both PTOA cartilage and bone damage [8], continuous intraarticular administrations demonstrated their efficacy on both tissue structures at all PTOA stages. One explanation for such differences can be exemplified by studies in inflammatory arthritis using *Ccr2-null* mice, which have led to the identification of a CCR2^+^ T-cell subpopulation that plays a role in downmodulating the inflammatory response during disease progression [40]. Therefore, a systemic approach to CCR2 targeting during PTOA, in addition to harboring potential side effects resulting from exposure to high drug doses, might not be ideal because it could suppress critical anti-inflammatory cells at certain disease stages and impact their activity.

DMM-induced synovitis has been reported after 4 weeks compared with sham controls [41]. Local CCR2 targeting was able to reduce synovial hyperplasia, indicating an anti-inflammatory action of RS504393. However, although visible in early PTOA, such changes became evident only at the severe PTOA stage (10 weeks). One explanation could be related to the increase in synovial thickness that occurs as PTOA evolves to more severe stages in the DMM model (Fig. 5C, 10 weeks of saline vs. 4 weeks of saline). Data from previous work suggested that although CCL12 levels were detected in joint tissues early after injury and seemed to mediate cartilage and bone damage in the DMM model, the other CCR2 ligands (CCL2, CCL7, CCL8, and CCL13) were not detectable in joint tissues up to 8 weeks post-injury; however, their protein levels were definitely high at the severe stage (12 weeks). Taken together, these data suggest that a more robust inflammatory response occurs at later stages in this model and might be mediated by a larger array of chemokines in addition to CCL12. Therefore, CCR2 targeting might be more effective on synovial changes at later PTOA stages. Further studies are needed to determine the efficacy of CCR2 targeting in mediating the full inflammatory response induced by injury, including subsynovial cell infiltration.

A slight improvement in joint structures was also visible with DF-µPL itself when compared with the saline-injected knees across all PTOA outcomes, although improvements were modest and never significant. This suggests that in addition to carrying therapeutic drugs, the µPLs could also provide a modest protective structural role in stabilizing the joint because of their shape, which is similar to that of small cushions allowing for deformability and deposition within the joint cavity along the cartilage and synovial linings [14]. However, further studies will be needed to verify this hypothesis and eventually identify the potential mechanism of action.

Importantly, none of the PTOA changes induced by injury were ameliorated or altered by the use of the free drug administered following the same timeline and the same dosage.

## Conclusion

To develop OA therapies that mirror the multitissue aspect of the disease, strategies for local targeting of cartilage and bone tissues are currently being developed [42–44]. In this context, we were able to reformulate the CCR2 antagonist RS504393 into homogeneous microparticles capable of releasing the therapeutic compound slowly and in a sustained fashion into PBS buffer, with 20% residual drug amounts after 21 days. When tested in vitro on chondrocytes, neither the loaded RS-µPLs or drug-free DF-µPLs affected cell viability, returning values well above 80% of those of the controls and comparable with free RS504393. This supported the in vivo studies aimed at assessing the intraarticular therapeutic potential of RS504393 on PTOA onset and progression.

Using concentrations 100-fold lower than previously reported for systemic treatments and with 1 injection every 3 weeks, RS-µPLs demonstrated beneficial effects on the AC lamina, preserving its structure at both early and late PTOA stages; improvement in extracellular matrix composition; protection from bone damage, decreasing subchondral bone thickness, osteophyte formation and delaying osteophyte ossification; and decreased hyperplasia of the synovial lining cells.

Taken together, our results not only validate the importance of local CCR2 targeting in preventing the structural damage of cartilage and bone following injury but also confirm our polymeric, biodegradable microplates as successful drug carriers for therapeutic purposes in arthritis.

## Supplementary Information

Below is the link to the electronic supplementary material.

Supplementary file1 (DOCX 10005 KB)

## Data Availability

The datasets generated during the current study are available from the corresponding author on reasonable request.

## Declarations

### Animal studies

All institutional and national guidelines for the care and use of laboratory animals were followed.

## References

[1] Brown TD, Johnston RC, Saltzman CL, Marsh JL, Buckwalter JA (2006). Posttraumatic osteoarthritis: a first estimate of incidence, prevalence, and burden of disease. J Orthop Trauma.

[2] Pelletier JP, Martel-Pelletier J, Rannou F, Cooper C (2016). Efficacy and safety of oral NSAIDs and analgesics in the management of osteoarthritis: Evidence from real-life setting trials and surveys. Semin Arthritis Rheum.

[3] Lin J, Zhang W, Jones A, Doherty M (2004). Efficacy of topical non-steroidal anti-inflammatory drugs in the treatment of osteoarthritis: meta-analysis of randomised controlled trials. BMJ.

[4] Garriga C, Goff M, Paterson E, Hrusecka R, Hamid B, Alderson J, Leyland K, Honeyfield L, Greenshields L, Satchithananda K (2021). Clinical and molecular associations with outcomes at 2 years after acute knee injury: a longitudinal study in the Knee Injury Cohort at the Kennedy (KICK). Lancet Rheumatol.

[5] Longobardi L, Jordan JM, Shi XA, Renner JB, Schwartz TA, Nelson AE, Barrow DA, Kraus VB, Spagnoli A (2018). Associations between the chemokine biomarker CCL2 and knee osteoarthritis outcomes: the Johnston County Osteoarthritis Project. Osteoarthritis Cartilage.

[6] Li L, Jiang BE (2015). Serum and synovial fluid chemokine ligand 2/monocyte chemoattractant protein 1 concentrations correlates with symptomatic severity in patients with knee osteoarthritis. Ann Clin Biochem.

[7] Appleton CT, Usmani SE, Pest MA, Pitelka V, Mort JS, Beier F (2015). Reduction in disease progression by inhibition of transforming growth factor alpha-CCL2 signaling in experimental posttraumatic osteoarthritis. Arthritis Rheumatol.

[8] Longobardi L, Temple JD, Tagliafierro L, Willcockson H, Esposito A, D'Onofrio N, Stein E, Li T, Myers TJ, Ozkan H (2017). Role of the C-C chemokine receptor-2 in a murine model of injury-induced osteoarthritis. Osteoarthritis Cartilage.

[9] Raghu H, Lepus CM, Wang Q, Wong HH, Lingampalli N, Oliviero F, Punzi L, Giori NJ, Goodman SB, Chu CR (2017). CCL2/CCR2, but not CCL5/CCR5, mediates monocyte recruitment, inflammation and cartilage destruction in osteoarthritis. Ann Rheum Dis.

[10] Sarafi MN, Garcia-Zepeda EA, MacLean JA, Charo IF, Luster AD (1997). Murine monocyte chemoattractant protein (MCP)-5: a novel CC chemokine that is a structural and functional homologue of human MCP-1. J Exp Med.

[11] Kapoor M, Martel-Pelletier J, Lajeunesse D, Pelletier JP, Fahmi H (2011). Role of proinflammatory cytokines in the pathophysiology of osteoarthritis. Nat Rev Rheumatol.

[12] Lisee C, Spang JT, Loeser R, Longobardi L, Lalush D, Nissman D, Schwartz T, Hu D, Pietrosimone B (2021). Tibiofemoral articular cartilage composition differs based on serum biochemical profiles following anterior cruciate ligament reconstruction. Osteoarthr Cartil.

[13] Willcockson H, Ozkan H, Chubinskaya S, Loeser RF, Longobardi L (2021). CCL2 induces articular chondrocyte MMP expression through ERK and p38 signaling pathways. Osteoarthr Cartil Open.

[14] Di Francesco M, Bedingfield SK, Di Francesco V, Colazo JM, Yu F, Ceseracciu L, Bellotti E, Di Mascolo D, Ferreira M, Himmel LE (2021). Shape-defined microplates for the sustained intra-articular release of dexamethasone in the management of overload-induced osteoarthritis. ACS Appl Mater Interfaces.

[15] Bedingfield SK, Colazo JM, Di Francesco M, Yu F, Liu DD, Di Francesco V, Himmel LE, Gupta MK, Cho H, Hasty KA (2021). Top-down fabricated microplates for prolonged, intra-articular matrix metalloproteinase 13 siRNA nanocarrier delivery to reduce post-traumatic osteoarthritis. ACS Nano.

[16] Di Francesco M, Primavera R, Summa M, Pannuzzo M, Di Francesco V, Di Mascolo D, Bertorelli R, Decuzzi P (2020). Engineering shape-defined PLGA microplates for the sustained release of anti-inflammatory molecules. J Control Release.

[17] Glasson SS, Askew R, Sheppard B, Carito B, Blanchet T, Ma HL, Flannery CR, Peluso D, Kanki K, Yang Z (2005). Deletion of active ADAMTS5 prevents cartilage degradation in a murine model of osteoarthritis. Nature.

[18] Glasson SS, Askew R, Sheppard B, Carito BA, Blanchet T, Ma HL, Flannery CR, Kanki K, Wang E, Peluso D (2004). Characterization of and osteoarthritis susceptibility in ADAMTS-4-knockout mice. Arthritis Rheum.

[19] Glasson SS, Blanchet TJ, Morris EA (2007). The surgical destabilization of the medial meniscus (DMM) model of osteoarthritis in the 129/SvEv mouse. Osteoarthr Cartil.

[20] McNulty MA, Loeser RF, Davey C, Callahan MF, Ferguson CM, Carlson CS (2011). A comprehensive histological assessment of osteoarthritis lesions in mice. Cartilage.

[21] Nagira K, Ikuta Y, Shinohara M, Sanada Y, Omoto T, Kanaya H, Nakasa T, Ishikawa M, Adachi N, Miyaki S (2020). Histological scoring system for subchondral bone changes in murine models of joint aging and osteoarthritis. Sci Rep.

[22] Little CB, Barai A, Burkhardt D, Smith SM, Fosang AJ, Werb Z, Shah M, Thompson EW (2009). Matrix metalloproteinase 13-deficient mice are resistant to osteoarthritic cartilage erosion but not chondrocyte hypertrophy or osteophyte development. Arthritis Rheum.

[23] Rowe MA, Harper LR, McNulty MA, Lau AG, Carlson CS, Leng L, Bucala RJ, Miller RA, Loeser RF (2017). Reduced osteoarthritis severity in aged mice with deletion of macrophage migration inhibitory factor. Arthritis Rheumatol.

[24] Mack M, Cihak J, Simonis C, Luckow B, Proudfoot AE, Plachy J, Bruhl H, Frink M, Anders HJ, Vielhauer V (2001). Expression and characterization of the chemokine receptors CCR2 and CCR5 in mice. J Immunol.

[25] Lefebvre E, Moyle G, Reshef R, Richman LP, Thompson M, Hong F, Chou HL, Hashiguchi T, Plato C, Poulin D (2016). Antifibrotic effects of the dual CCR2/CCR5 antagonist cenicriviroc in animal models of liver and kidney fibrosis. PLoS ONE.

[26] Menne J, Eulberg D, Beyer D, Baumann M, Saudek F, Valkusz Z, Wiecek A, Haller H, Emapticap Study G (2017). C-C motif-ligand 2 inhibition with emapticap pegol (NOX-E36) in type 2 diabetic patients with albuminuria. Nephrol Dial Transplant.

[27] Ninichuk V, Clauss S, Kulkarni O, Schmid H, Segerer S, Radomska E, Eulberg D, Buchner K, Selve N, Klussmann S (2008). Late onset of Ccl2 blockade with the Spiegelmer mNOX-E36-3'PEG prevents glomerulosclerosis and improves glomerular filtration rate in db/db mice. Am J Pathol.

[28] Ishihara S, Obeidat AM, Wokosin DL, Ren D, Miller RJ, Malfait AM, Miller RE (2021). The role of intra-articular neuronal CCR2 receptors in knee joint pain associated with experimental osteoarthritis in mice. Arthritis Res Ther.

[29] Bellotti E, Schilling AL, Little SR, Decuzzi P (2021). Injectable thermoresponsive hydrogels as drug delivery system for the treatment of central nervous system disorders: a review. J Control Release.

[30] Primavera R, Bellotti E, Di Mascolo D, Di Francesco M, Wang J, Kevadiya BD, De Pascale A, Thakor AS, Decuzzi P (2021). Insulin granule-loaded microplates for modulating blood glucose levels in type-1 diabetes. ACS Appl Mater Interfaces.

[31] Bodick N, Lufkin J, Willwerth C, Kumar A, Bolognese J, Schoonmaker C, Ballal R, Hunter D, Clayman M (2015). An intra-articular, extended-release formulation of triamcinolone acetonide prolongs and amplifies analgesic effect in patients with osteoarthritis of the knee: a randomized clinical trial. J Bone Joint Surg Am.

[32] Moore TL, Cook AB, Bellotti E, Palomba R, Manghnani P, Spano R, Brahmachari S, Di Francesco M, Palange AL, Di Mascolo D (2022). Shape-specific microfabricated particles for biomedical applications: a review. Drug Deliv Transl Res.

[33] Simon-Vazquez R, Tsapis N, Lorscheider M, Rodriguez A, Calleja P, Mousnier L, de Miguel VE, Gonzalez-Fernandez A, Fattal E (2022). Improving dexamethasone drug loading and efficacy in treating arthritis through a lipophilic prodrug entrapped into PLGA-PEG nanoparticles. Drug Deliv Transl Res.

[34] Willcockson H, Ozkan H, Chubinskaya S, Loeser RF, Longobardi L. CCL2 Induces Articular Chondrocyte MMP expression through ERK and p38 Signaling Pathways. Osteoarthr Cartil Open. 2021;100136. 10.1016/j.ocarto.2020.100136 PMC9718225 36475068

[35] Teeple E, Jay GD, Elsaid KA, Fleming BC (2013). Animal models of osteoarthritis: challenges of model selection and analysis. AAPS J.

[36] Burr DB (2005). Increased biological activity of subchondral mineralized tissues underlies the progressive deterioration of articular cartilage in osteoarthritis. J Rheumatol..

[37] Kouri JB, Aguilera JM, Reyes J, Lozoya KA, Gonzalez S (2000). Apoptotic chondrocytes from osteoarthrotic human articular cartilage and abnormal calcification of subchondral bone. J Rheumatol.

[38] Zamli Z, Robson Brown K, Tarlton JF, Adams MA, Torlot GE, Cartwright C, Cook WA, Vassilevskaja K, Sharif M (2014). Subchondral bone plate thickening precedes chondrocyte apoptosis and cartilage degradation in spontaneous animal models of osteoarthritis. Biomed Res Int.

[39] Xing Z, Lu C, Hu D, Yu YY, Wang X, Colnot C, Nakamura M, Wu Y, Miclau T, Marcucio RS (2010). Multiple roles for CCR2 during fracture healing. Dis Model Mech.

[40] Bruhl H, Cihak J, Schneider MA, Plachy J, Rupp T, Wenzel I, Shakarami M, Milz S, Ellwart JW, Stangassinger M (2004). Dual role of CCR2 during initiation and progression of collagen-induced arthritis: evidence for regulatory activity of CCR2+ T cells. J Immunol.

[41] Jackson MT, Moradi B, Zaki S, Smith MM, McCracken S, Smith SM, Jackson CJ, Little CB (2014). Depletion of protease-activated receptor 2 but not protease-activated receptor 1 may confer protection against osteoarthritis in mice through extracartilaginous mechanisms. Arthritis Rheumatol.

[42] Laroui H, Grossin L, Leonard M, Stoltz JF, Gillet P, Netter P, Dellacherie E (2007). Hyaluronate-covered nanoparticles for the therapeutic targeting of cartilage. Biomacromol.

[43] Sandker MJ, Petit A, Redout EM, Siebelt M, Muller B, Bruin P, Meyboom R, Vermonden T, Hennink WE, Weinans H (2013). In situ forming acyl-capped PCLA-PEG-PCLA triblock copolymer based hydrogels. Biomaterials.

[44] Zhang Y, Wei L, Miron RJ, Shi B, Bian Z (2015). Anabolic bone formation via a site-specific bone-targeting delivery system by interfering with semaphorin 4D expression. J Bone Miner Res Off J Am Soc Bone Miner Res.

