# Supplementary material for: Sustained inhibition of CC-chemokine receptor-2 via intraarticular deposition of polymeric microplates in post-traumatic osteoarthritis
Source: Drug Deliv Transl Res. 2022 Sep 15;13(2):689–701. doi: 10.1007/s13346-022-01235-1 (PMC9794532; doi:10.1007/s13346-022-01235-1)
Supplement: Supplementary file 1 — Supplementary file1 (DOCX 10005 KB) [file 13346_2022_1235_MOESM1_ESM.docx]

**Supplementary Information**

**Sustained Inhibition of CC-chemokine Receptor-2 via intra-articular deposition of polymeric microPlates in Post-Traumatic Osteoarthritis**

***Huseyin Ozkan^1^, *Martina Di Francesco^2^, Helen Willcockson^1^, José Valdés-Fernández^1,3^, Valentina Di Francesco^2^, Froilán Granero-Moltó^3,4,5,6^, Felipe Prósper^3,5,6,7,8^,** ^♣︎^**Paolo Decuzzi^2^ and** ^♣︎^**Lara Longobardi^1^**

*^1^Division of Rheumatology, Allergy and Immunology and the Thurston Arthritis Research Center, University of North Carolina-Chapel Hill, NC*

*^2^Laboratory of Nanotechnology for Precision Medicine, Fondazione Istituto Italiano di Tecnologia, Genova, Italy.*

*^3^Cell Therapy Area, Clínica Universidad de Navarra, Pamplona, Spain.*

*^4^Department of Orthopedic Surgery and Traumatology, Clínica Universidad de Navarra, Pamplona, Spain.*

*^5^Program of Regenerative Medicine, Center for Applied Medical Research (CIMA), Universidad de Navarra, Pamplona, Spain.*

*^6^Instituto de Investigacion Sanitaria de Navarra (IdiSNA), Pamplona, Spain*

*^7^Department of Hematology, Clínica Universidad de Navarra, Pamplona, Spain.*

*^8^Program of Hemato-Oncology, Center for Applied Medical Research (CIMA), Universidad de Navarra, Pamplona, Spain.*

**Address all correspondence and requests for reprints to:**

Lara Longobardi, PhD, Division of Rheumatology, Allergy and Immunology, University of North Carolina at Chapel Hill, 3300 Thurston Bowels Bldg, Campus Box 7280, Chapel Hill, NC 27599. Phone: (919) 843-4727. Fax: (919) 966-1739. E-mail: [lara_longobardi@med.unc.edu](mailto:lara_longobardi@med.unc.edu). ORCID ID: 0000-0002-0156-847X

* HO and MDF contributed equally to this work

^♣︎^ LL and PD share the senior authorship

**Supplementary Methods**

**Histopathologic assessment of arthritis** Dissected knees were fixed in 4% paraformaldehyde, overnight at RT. Following removal of fixative and rinsing in PBS, knees were decalcified with Immunocal (StatLab, McKinney, TX) for 5-7 days, embedded in paraffin, and frontal sections (6µm) were cut through the entire joint. Sections at 70µm intervals were stained with Safranin-O/Fast Green and images taken with an Olympus BX51 microscope and a DP71 camera. For OA grading, we used 2 different semiquantitative scoring systems, described by McNulty et al 2007, the Articular Cartilage Structure (ACS) score and the Safranin-O staining score (Saf-O) [20]. Compared to the OARSI score system, designed to rapidly identify the site within the joint that contains the most severe lesions, the combination of ACS and Saf-O scores provide in-depth information regarding changes within the lesions, for both articular cartilage structure and extracellular matrix integrity. The ACS focuses on AC structure, identifying fibrillations and cleft in the structure, while the Saf-O is more tailored at identifying changes within the cell compartment and/or in the extracellular matrix For both scoring systems, two adjacent midcoronal and posterior sections were stained with H & E (for ACS) or Safranin-O and lesions are identified within the 4 compartments (medial and lateral tibial plateau and femoral condyles); In both ACS and Saf-O scores, lesions are scored on a 0-12 scale as described by McNulty et al., where zero is defined as normal, reflecting a smooth articular surface (ACS) or uniform staining throughout the articular cartilage (Saf-O), while 12 reflects fibrillation/cleft/loss of cartilage (ACS) or complete loss of staining in both cells and matrix (Saf-O) involving the full thickness of articular cartilage with an extension higher than two third of the plateau/condyle [20].

**Osteophyte size and maturity scoring**. For osteophyte grading, we used the histological scoring system developed by Little et al, developed to score both osteophyte size (from 1 to 3) and osteophyte maturity (from 1 to 3), with the latter reflecting the osteophyte tissue composition [22]. Osteophyte grading was performed on Safranin-O/Fast-Green sections to obtain the OARSI score. Because osteophytes usually form close to areas of cartilage degradation, in the DMM they are predominately localized on the medial tibial plateau, therefore only sections from this region were used for osteophyte grading. Briefly, an osteophyte size score is obtained on assigning a score based on the size of osteophytes compared to adjacent cartilage (0 = none, 1 = small ~ the same thickness as the adjacent cartilage, 2 = medium ~ 1–3 × the thickness as the adjacent cartilage, 3 = large >3 × the thickness as the adjacent cartilage). The osteophyte maturity score reflects the tissue composition of osteophytes and is assessed by assigning a score as following: 0 = none, 1 = predominantly cartilaginous, 2 = mixed cartilage and bone with active vascular invasion and endochondral ossification, 3 = predominantly bone [22].

Both osteophyte size and maturity scores were obtained from coded digital images of the same location of the anterior-medial tibia in each animal.

We also quantified the area of cartilage within the osteophytes using Image-J [21]. The osteophyte images from samples stained with Saf-O from all groups were encircled and analyzed as a separate images. These new images were used to calculate the total area of the osteophyte as well as the area of Saf-O staining, and measures were expressed as ratio between Saf-O Area/Total area.

**Supplementary Results**

**
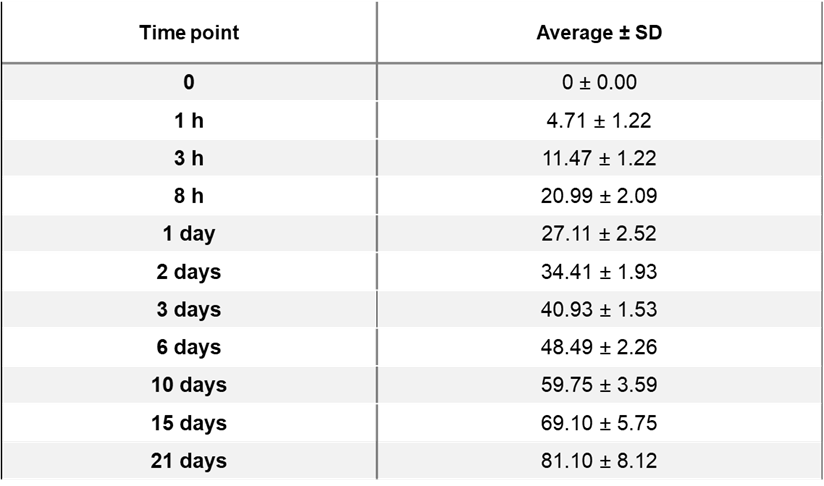
**

**Supplementary Table S1**. Average percentage and standard deviation (SD) of RS504393 released from µPLs for each considered time point under confined microenvironment.

**
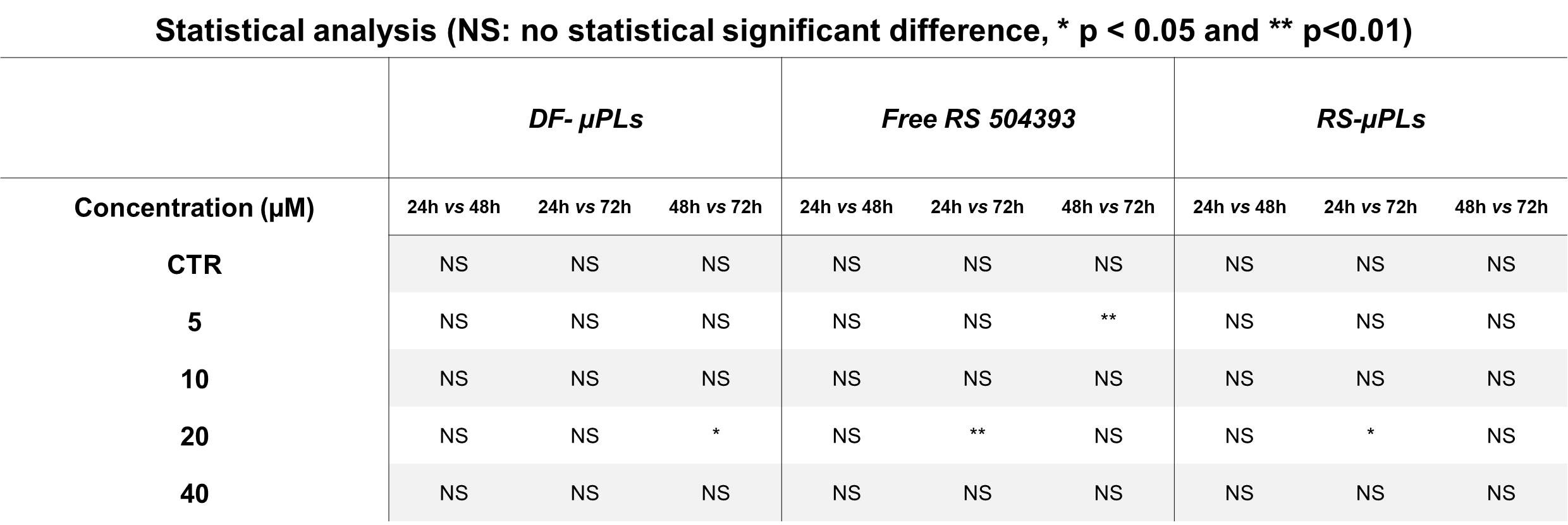
**

**Supplementary Table S2**. Statistically significant differences for all the experimental groups of **Fig.2**. Significance corresponds to a p-value smaller than 0.05.

**Supplementary Table S3**. Effect of sustained intra-articular release of RS504393 (4wks and 10wks post-DMM) on OA assessment (N=6) in DMM knee joints. Data presented shows mean differences, 95% CI (in parentheses) between groups at the corresponding time point.


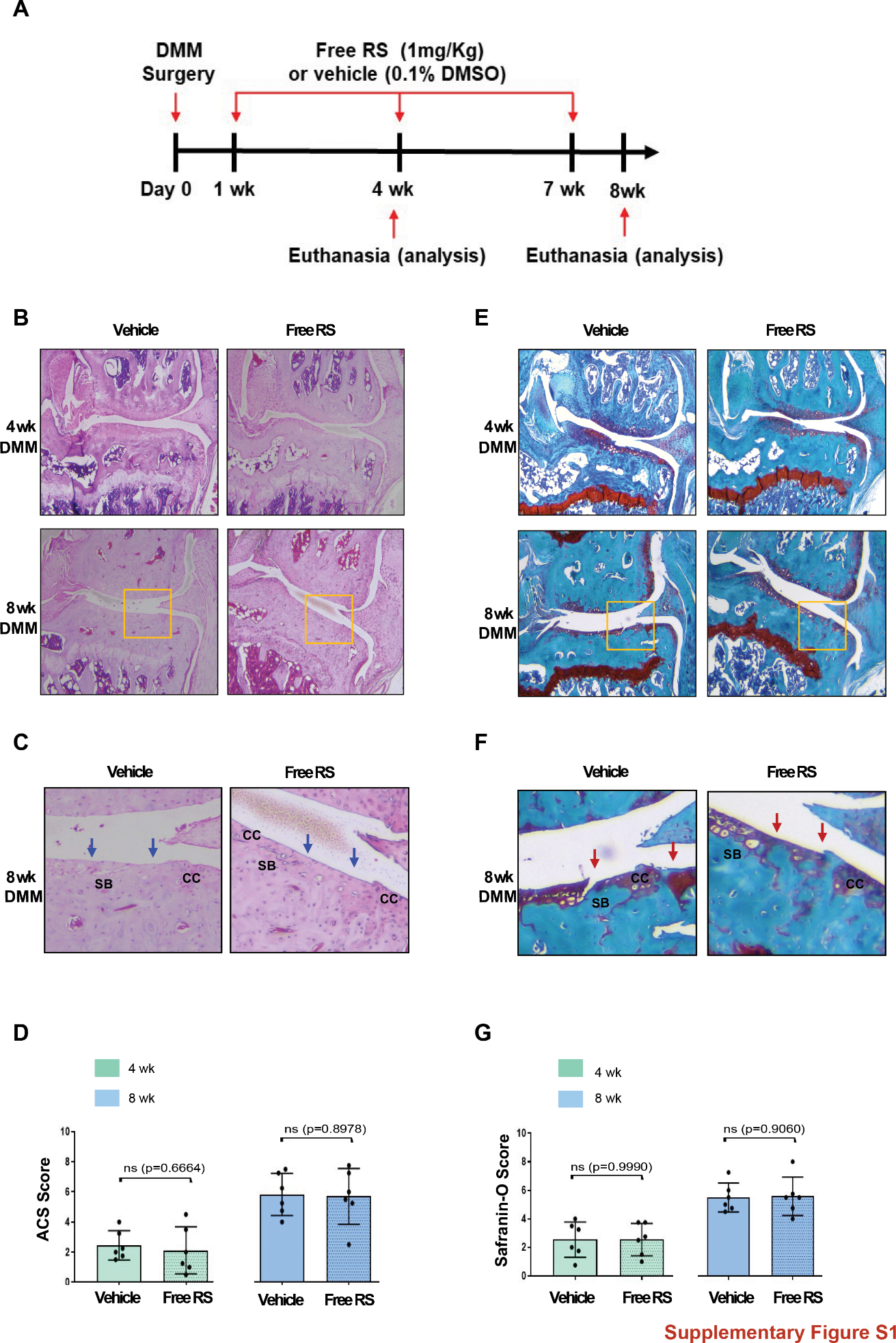


**Supplementary Fig. S1. Histopathological evaluation of cartilage structure (ACS score) and extracellular matrix (Safranin-O Score) of DMM mouse knees, following intra-articular administration of free RS504393 or vehicle.** (**A**) Schematic view of timeline for treatment and data evaluation. (**B**) H&E staining of the knee of DMM mice, showing the medial compartment of joints injected intra-articularly with free RS504393 or vehicle, at the indicated times following surgery; images are representative of *n*=6 for each time point. (**C**) Magnified images relative to the yellow square indicated in Suppl. Fig. S1B; magnifications of the DMM knees are from the severe PTOA stage (10wk) and show regions of absence of cartilage in the both the veichle and fre-RS injected samples (blue arrows). (**D**) ACS semiquantitative score (0-12 scale) of DMM knees at the time points indicated, reflecting the structure of the articular surface (lamina); the grading accounts for both the depth and extension of the damage. Results are expressed as average of 4 quadrants (medial and lateral tibial plateau, medial and lateral femoral condyles); *n*=6 mice for each experimental point. (**E**) Safranin-O/Fast green staining of the knee of DMM mice, showing the medial compartment of joints injected intra-articularly with free RS504393 or vehicle, at the indicated times following surgey; images are representative of *n*=6 for each time points. (**F**) Magnified images relative to the yellow square indicated in Suppl. Fig. S1E; magnifications of the DMM knees are from the severe PTOA stage (10wk) and show regions with loss of Safranin-O staining, in both the vehicle and free-RS injected samples (red arrows). (**G**) Safranin-O semiquantitative score (0-12 scale) of DMM knees at the time point indicated, reflecting loss of EM; the grading accounts for both the depth and extension of the damage. Results are expressed as average of 4 quadrants as described above; *n*=6 mice for each experimental point. The graphs represent the mean ± standard deviation. Student’s unpaired t-tests is indicated in each graph at each time point. Scale bars of the images are 100 µm.


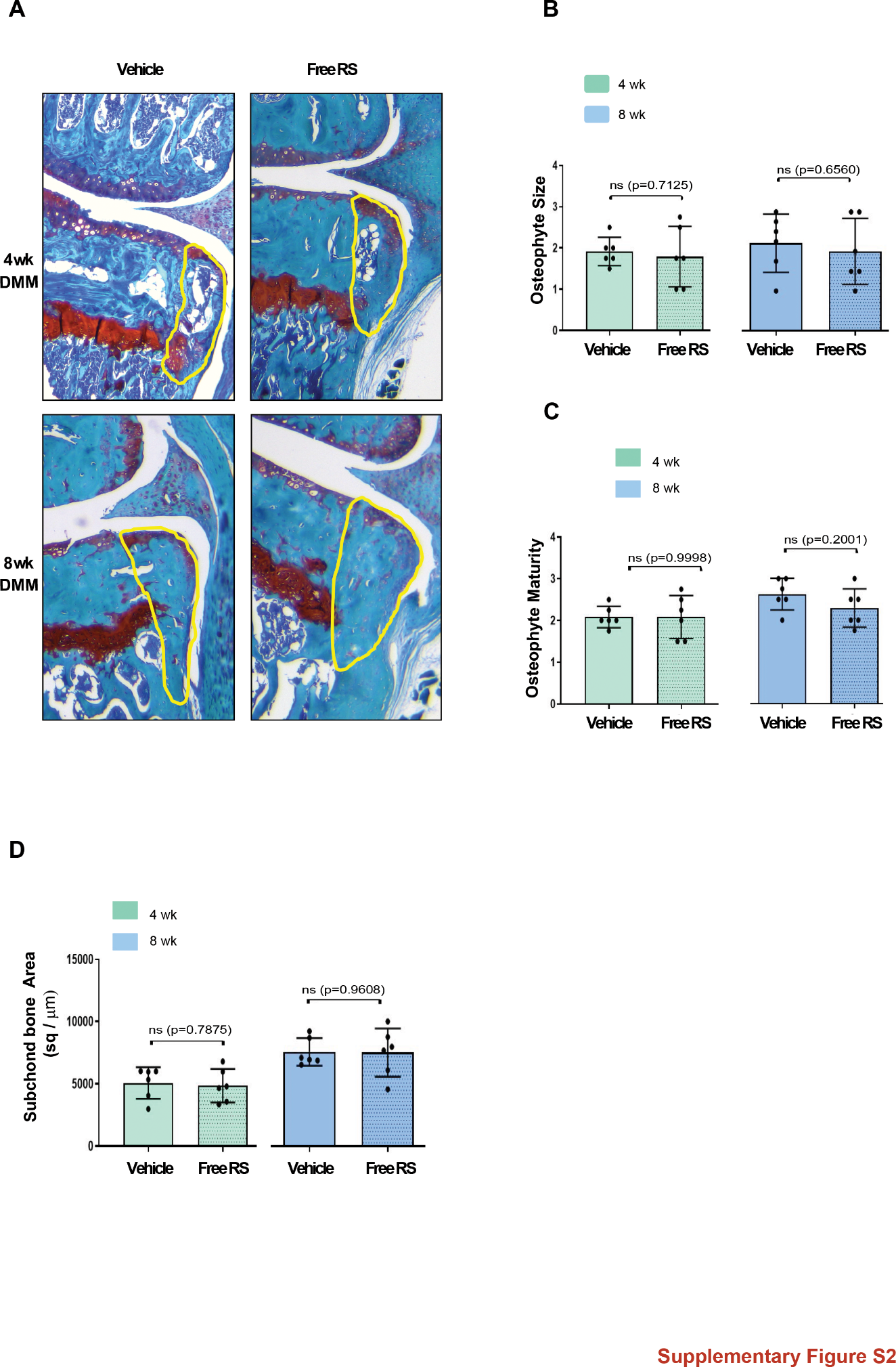


**Supplementary Fig. S2 Osteophyte assessment and subchondral bone quantification of DMM mouse knees, following intra-articular administration of free RS504393 or vehicle.** (**A**) Safranin-O/Fast green staining of osteophyte formations in the medial tibial compartment of DMM mice injected intra-articularly with free RS504393 or vehicle, at the indicated times; osteophytes have been circled in the images, representing new cartilage/bone formations emerging at the level of the lateral tibia; images are representative of *n*=6 for each of the experimental points described. (**B**) The osteophyte size score at the time points indicated represents a semiquantitative grading of the size of the new formed osteophytes (scale 0-3). Results are expressed as average of 4 quadrants (medial and lateral tibial plateau, medial and lateral femoral condyles); *n*=6 mice for each experimental point. (**C**) The osteophyte maturity score (scale 0-3) at the time point indicated represents a semiquantitative grading of the amount of bone tissue in the osteophytes. Results are expressed as average of 4 quadrants (medial and lateral tibial plateau, medial and lateral femoral condyles); *n*=6 mice for each experimental point. (**D**) Quantification of area of cartilage in the osteophytes is calculated by histomorphometric analysis and expressed as percentage of the total osteophyte area, at each experimental time point; *n*=6 mice for each experimental point. (**E**) Quantification of the subchondral plate area (sq/ µm) of the tibia medial plateau of DMM knees by histomorphometric analysis at the time point indicated; anatomically, the subchondral bone is defined between the calcified cartilage and the trabecular bone that surrounds the bone marrow regions; *n*=6 mice for each experimental point.

The graphs represent the mean ± standard deviation. Student’s unpaired t-tests is indicated in each graph at each time point. Scale bars of the images are 100 µm.


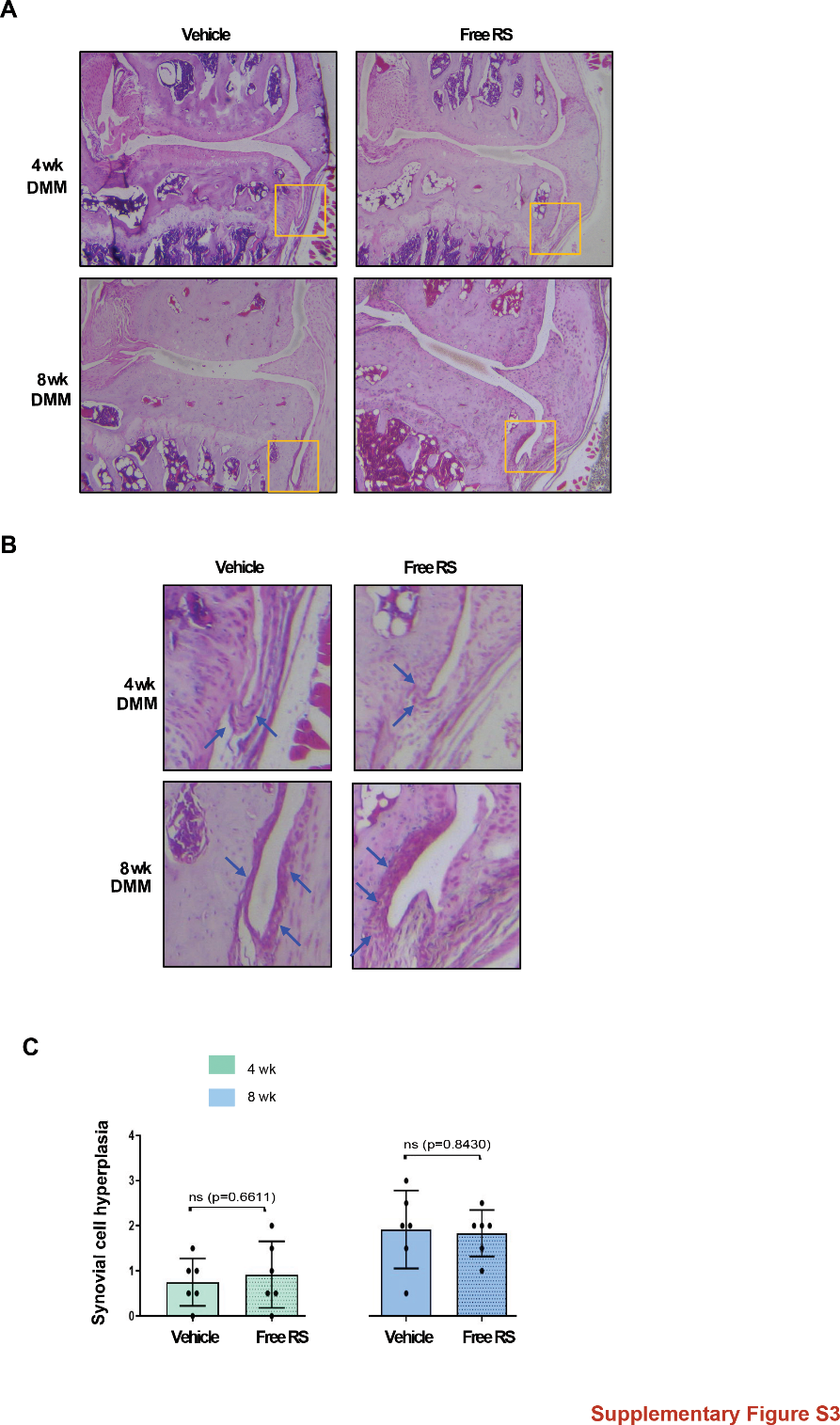


**Supplementary Fig. S3. Synovial hyperplasia assessment of DMM mouse knees, following intra-articular administration of free RS504393 or vehicle.** **(A)** H&E staining of the synovial lining cell of DMM mice, injected intra-articularly with free RS504393 or vehicle, at the indicated times following surgery; images are representative of *n*=6 for each time point. (**B**) Magnified images relative to the yellow square indicated in Suppl. Fig. S3A; images show the thickness of the synovium in both vehicle samples and fre-RS injected samples (blue arrows). (**C**) Synovial hyperplasia scores (scale 0-3) at the time point indicated, reflecting the thickness of the synovium (number of cell layers in the thickest point). Scores reflect the highest grade of the medial and lateral tibial plateau, medial and lateral femoral condyles; *n=6* mice for each experimental point. The graphs represent the mean ± standard deviation. Student’s unpaired t-tests is indicated in each graph at each time point. Scale bars of the images are 100 µm.


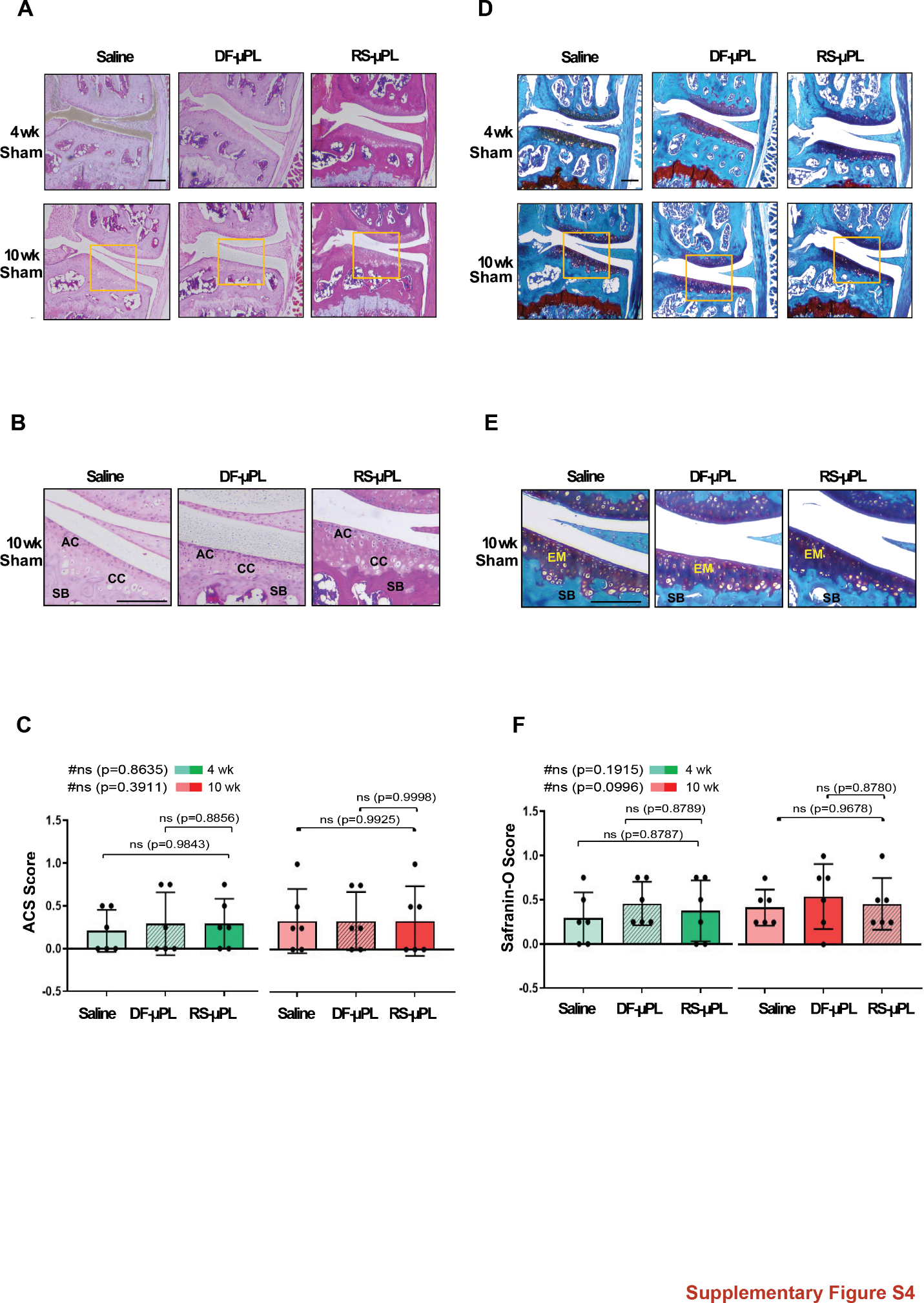


**Supplementary Fig. S4. Histopathological evaluation of cartilage structure (ACS score) and extracellular matrix (Safranin-O Score) of Sham mouse knees, following intra-articular administration of RS-μPL, DF- μPL or saline solution.** (**A**) H&E staining of the knee of Sham mice, showing the medial compartment of Sham joints injected intra-articularly with saline, drug free μPLs (DF-μPL) or RS504393-loaded μPLs (RS-μPL), at the indicated times following surgery; images are representative of *n*=6 for each time point. (**B**) Magnified images relative to the yellow square indicated in Suppl. Fig. S4A; magnifications of the Sham knees are from the severe PTOA stage (10wk) and show a smooth articular cartilage surface in all samples. (**C**) ACS semiquantitative score (0-12 scale) of Sham knees at the time points indicated, reflecting the structure of the articular surface (lamina); the grading accounts for both the depth and extension of the damage. Results are expressed as average of 4 quadrants (medial and lateral tibial plateau, medial and lateral femoral condyles); *n*=6 mice for each experimental point. (**D**) Safranin-O/Fast green staining of the knee of Sham joints injected intra-articularly with Saline, drug free μPLs (DF-μPL) or RS504393-loaded μPLs (RS-μPL), at the indicated times following surgey; images are representative of *n*=6 for each time points. (**E**) Magnified images relative to the yellow square indicated in Suppl. Fig. S4D; magnifications of the DMM knees are from the severe PTOA stage (10wk) and show a uniform Safranin-O staining in all samples across the whole tibial plateau. (**F**) Safranin-O semiquantitative score (0-12 scale) of Sham knees at the time point indicated. Results are expressed as average of 4 quadrants as described above; *n*=6 mice for each experimental point. The graphs represent the mean ± standard deviation. # indicates one-way ANOVA at each time point; multiple comparison values by Tukey’s post hoc test are indicated in each graph. Scale bars of the images are 100 µm.


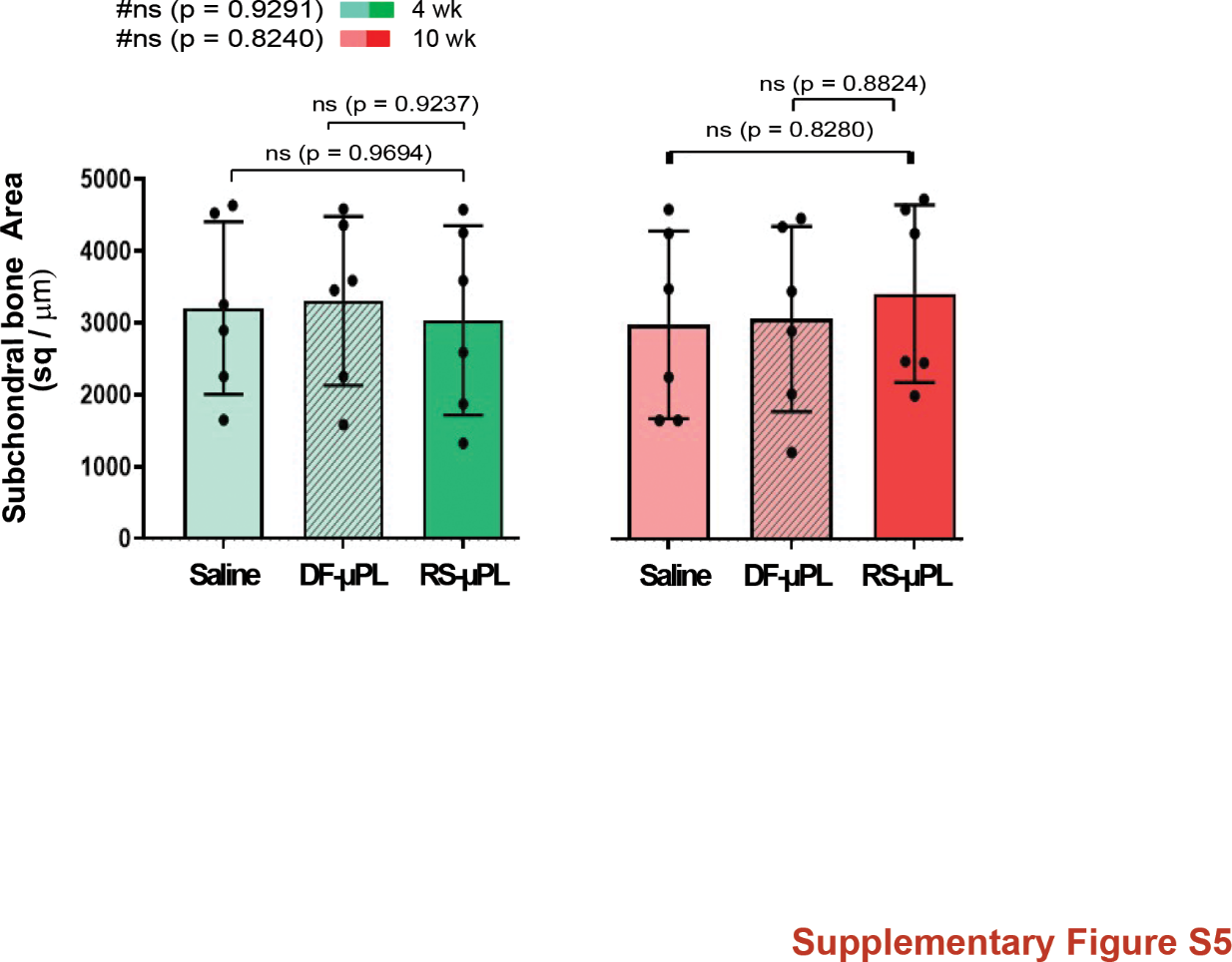


**Supplementary Figure S5. Subchondral bone quantification of Sham mouse knees, following intra-articular administration of RS-μPL, DF- μPL or Saline solution.** Quantification of the subchondral plate area (sq/µm) of the tibia medial plateau of Sham knees by histomorphometric analysis at the time point indicated; anatomically, the subchondral bone is defined between the calcified cartilage and the trabecular bone that surrounds the bone marrow regions; *n*=6 mice for each experimental point. The graphs represent the mean ± standard deviation. # indicates one-way ANOVA at each time point; multiple comparison values by Tukey’s post hoc test are indicated in each graph. Scale bars of the images are 100 µm.


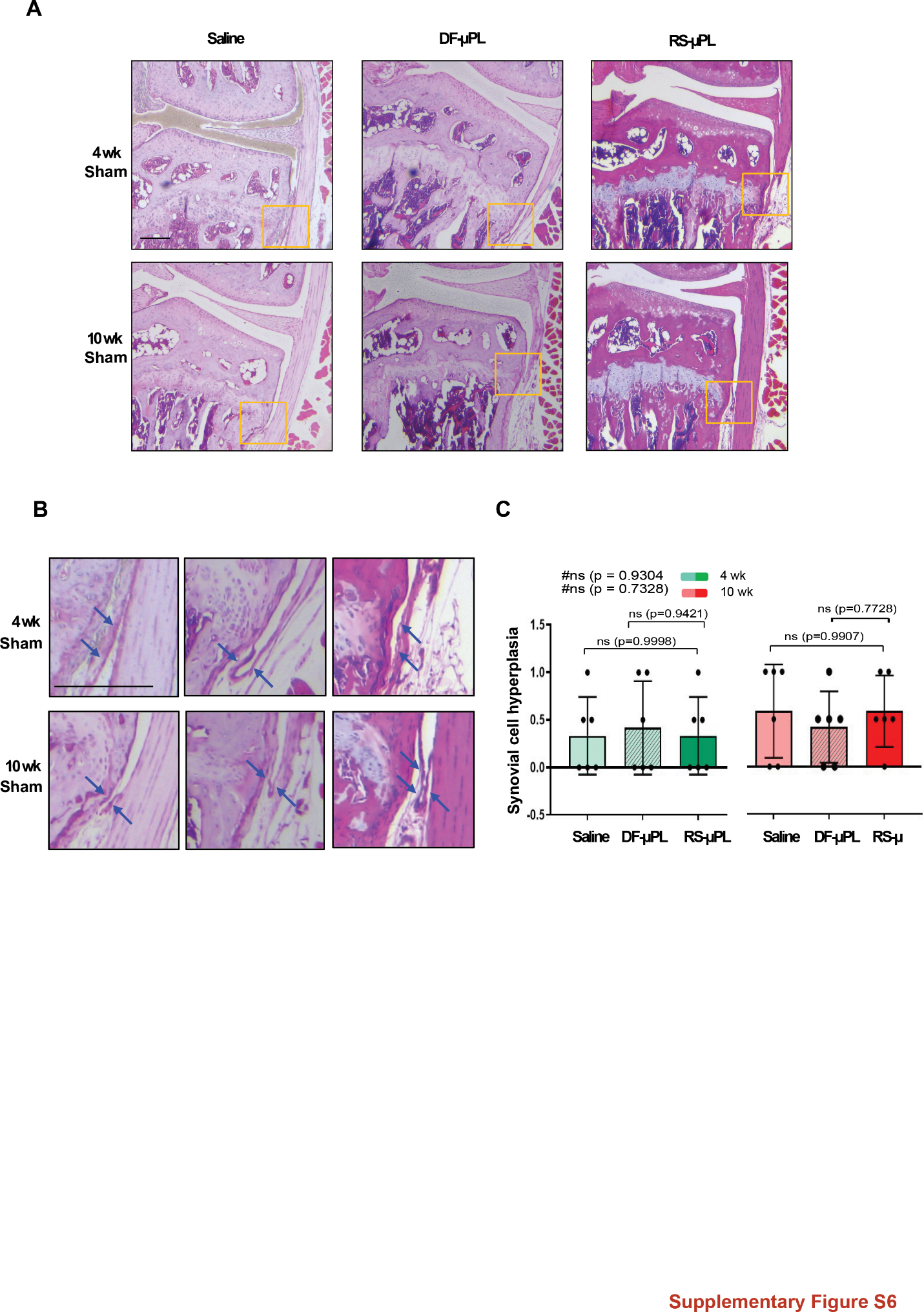


**Supplementary Fig. S6. Synovial hyperplasia assessment of Sham mouse knees, following intra-articular administration of RS-μPL, DF- μPL or Saline solution. (A)** H&E staining of the synovial lining cell of Sham mice, injected intra-articularly with saline, drug free μPLs (DF-μPL) or RS504393-loaded μPLs (RS-μPL), at the indicated times following surgery; images are representative of *n*=6 for each time point. (**B**) Magnified images relative to the yellow square indicated in Suppl. Fig. S6A; the thickness of the synovium did not reach a 3-cell thickness in any of the samples analyzed (blue arrows). (**C**) Synovial hyperplasia scores (scale 0-3) at the time point indicated, reflecting the thickness of the synovium (number of cell layers in the thickest point). Scores reflect the highest grade of the medial and lateral tibial plateau, medial and lateral femoral condyles; *n=6* mice for each experimental point. The graphs represent the mean ± standard deviation. # indicates one-way ANOVA at each time point; multiple comparison values by Tukey’s post hoc test are indicated in each graph. Scale bars of the images are 100 µm.
